# Transport mechanism and structural pharmacology of human urate transporter URAT1

**DOI:** 10.1038/s41422-024-01023-1

**Published:** 2024-09-09

**Authors:** Yaxin Dai, Chia-Hsueh Lee

**Affiliations:** Department of Structural Biology, St. Jude Children’s Research Hospital, Memphis, TN USA

**Keywords:** Cryoelectron microscopy, Mechanisms of disease

## Abstract

Urate is an endogenous product of purine metabolism in the liver. High urate levels in the blood lead to gout, a very common and painful inflammatory arthritis. Excreted urate is reabsorbed in the kidney mainly by URAT1 antiporter, a key target for anti-gout drugs. To uncover the mechanisms of urate transport and drug inhibition, we determined cryo-EM structures of human URAT1 with urate, counter anion pyrazinoate, or anti-gout drugs of different chemotypes — lesinurad, verinurad, and dotinurad. We captured the outward-to-inward transition of URAT1 during urate uptake, revealing that urate binds in a phenylalanine-rich pocket and engages with key gating residues to drive the transport cycle. In contrast to the single binding site for urate, pyrazinoate interacts with three distinct, functionally relevant sites within URAT1, a mechanism that has not yet been observed in other anion antiporters. In addition, we found that while all three drugs compete with substrates and halt the transport cycle, verinurad and dotinurad further hijack gating residues to achieve high potency. These insights advance our understanding of organic anion transport and provide a foundation for designing improved gout therapeutics.

## Introduction

Urate is an important endogenous metabolite in humans. Generated by the liver as the end product of purine metabolism, urate is excreted by the kidney and intestine.^1^ Excess urate in the blood can form crystals that accumulate in the joints, leading to inflammation.^2^ This type of inflammatory arthritis, also known as gout, often causes sudden and intense pain, debilitating mobility, and deterioration of life quality.^3^ It is estimated that gout affects about 1%–7% of the population across different countries,^4^ causing substantial healthcare costs and economic burdens. High levels of urate in the blood are also associated with hypertension.^5^

Urate homeostasis is tightly regulated by its secretion and reabsorption in the kidney. Among the several renal transporters involved in urate flux, urate transporter 1 (URAT1) is the primary one responsible for urate reabsorption.^6–8^ Mutations in the human URAT1 gene decrease blood urate levels and increase urate excretion, leading to hypouricemia and related kidney diseases.^6,9–12^ URAT1, or SLC22A12, belongs to the organic anion transporter (OAT) subgroup of the solute carrier family 22 (SLC22).^6,13^ It functions as an antiporter, moving intracellular organic anions or chloride ions to drive the uptake of extracellular urate from the renal tubule lumen into the cells.^6^ URAT1 can export endogenous lactate and nicotinate, or exogenous pyrazinoate (a metabolite from the front-line anti-tuberculosis drug pyrazinamide)^6,14^ in exchange for urate; both nicotinate and pyrazinoate are known anti-uricosuric agents that potentiate urate absorption in the kidney.^14–17^

URAT1 is also an established therapeutic target for the treatment of hyperuricemia and gout. Uricosuric agents, such as lesinurad, verinurad, and dotinurad,^18–20^ inhibit URAT1 activity to prevent urate reabsorption and accumulation in the blood. Lesinurad has been approved for treating gout in the United States. Its derivative, verinurad, exhibits substantially higher potency and specificity and is currently undergoing clinical trials. A third drug, dotinurad, which is structurally divergent from lesinurad, has high affinity and selectivity for URAT1 and has been approved in Japan.

Despite the high physiological and medical relevance of URAT1, and the intense research since the gene was cloned over two decades ago,^6^ its transport mechanism and structural pharmacology are still poorly understood. Recent advances have revealed the overall architecture of SLC22 family transporters and provided insights into ligand recognition and transport.^21–26^ Nevertheless, compared to the uniporter type of SLC22 (e.g., OCT1–3), the mechanism of the antiporter type of SLC22, including URAT1, remains less clear. Structures of a related urate transporter OAT1 (SLC22A6) showed how OAT1 interacts with its exogenous substrate para-aminohippuric acid, and counter anion α-ketoglutarate.^21,22^ However, URAT1 has a distinct substrate selectivity and drug sensitivity profile among the SLC22 family; for instance, URAT1 does not transport para-aminohippuric acid or α-ketoglutarate.^6^ Moreover, all available OAT1 structures present an inward-facing conformation and do not have urate bound. Thus, how urate is imported by renal SLC22 antiporters and how intracellular anions are leveraged to facilitate urate uptake remain open questions. The molecular mechanism by which anti-gout drugs inhibit this process in URAT1 is also unknown.

In this study, we report 10 cryo-electron microscopy (cryo-EM) structures of human URAT1, captured in different functional states of its transport cycle. The structures unveil the principles of urate recognition and translocation by a transporter for the first time. Together with functional studies, we identify substrate sites for urate and counter organic anions, reveal the mechanisms of urate transport and drug inhibition, and suggest a model for the URAT1 antiporter cycle.

## Results

### Structural determination of human URAT1

URAT1 is a relatively small protein (~60 kDa), making cryo-EM particle alignment difficult. To facilitate structure determination of URAT1, we increased the particle size by fusing a maltose-binding protein (MBP) to its N-terminus and an MBP-specific, designed ankyrin repeat protein (DARPin)^27^ to its C-terminus. This strategy has enabled the structure determination of other small transporters.^28,29^

**Fig. 1:**
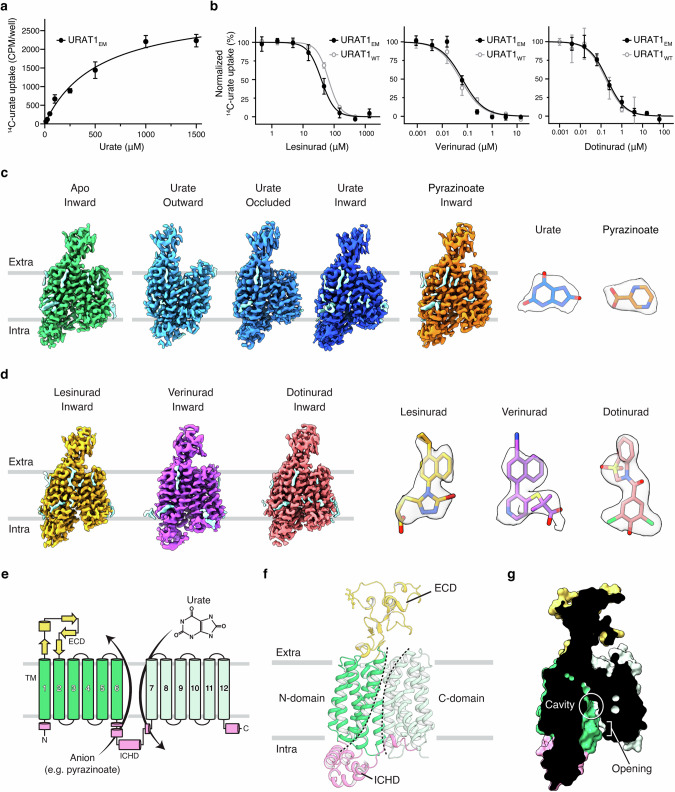
Cryo-EM structures of human URAT1 in different functional states. **a** ^14^C-urate uptake in HEK293 cells expressing the cryo-EM construct of URAT1 (URAT1_EM_). K_M_ = 533.2 µM. Data are shown as mean ± SD; *n* = 4 biological replicates. **b** Inhibition profiles of URAT1_EM_ and WT URAT1 (URAT1_WT_) by different drugs: left, lesinurad (IC_50_ for URAT1_EM_ = 39.0 µM, for URAT1_WT_ = 66.3 µM); center, verinurad (IC_50_ for URAT1_EM_ = 0.063 µM, for URAT1_WT_ = 0.053 µM); right, dotinurad (IC_50_ for URAT1_EM_ = 0.19 µM, for URAT1_WT_ = 0.17 µM). Data are shown as mean ± SD; *n* = 3 biological replicates. In **a**, **b**, uptake buffer: 20 mM HEPES pH 7.4, 125 mM sodium gluconate, 4.8 mM potassium gluconate, 1.2 mM monobasic potassium phosphate, 1.2 mM magnesium sulfate, 1.3 mM calcium gluconate, and 5.6 mM glucose. **c** Cryo-EM density maps of URAT1 in the apo and substrate-bound states. Representative densities of urate and pyrazinoate are shown on the right. Putative lipid densities are in light cyan. **d** Cryo-EM density maps of URAT1 in the drug-bound states. Densities for lesinurad, verinurad, and dotinurad are shown on the right. Putative lipid densities are in light cyan. **e** Diagram of URAT1 topology. ECD, extracellular domain. **f** Structure of URAT1 in the apo state. **g** Slice view of URAT1 in the apo state, showing the central cavity and opening to the intracellular side.

We initially expressed and purified the wild-type (WT) human URAT1 tagged with MBP and DARPin. However, the yield was relatively low, and the purified protein was prone to aggregation. We noticed that a closely related homolog of URAT1, OAT4 (SLC22A11), exhibited much better biochemical behavior (Supplementary information, Fig. S1a). We hypothesized that transplanting a segment from OAT4 might improve URAT1’s behavior, and therefore replaced a part of the intracellular loop of URAT1 (residues 280–343) with the corresponding sequence from OAT4 (residues 277–339). The resulting construct showed improved yield and protein homogeneity (Supplementary information, Fig. S1a), but formed dimers through its extracellular domain in cryo-EM grids (Supplementary information, Fig. S1b). This dimerization was likely induced by the high protein concentration used to prepare the grids, as the construct migrated as a monomer in size-exclusion chromatography. The dimeric particles had a suboptimal distribution and preferred orientation in the vitreous ice of the cryo-EM grids, hindering structure determination. To overcome this hurdle, we replaced a short loop of the extracellular domain of URAT1 (residues 60–65) with the corresponding sequence from OAT4 (residues 60–62) (Supplementary information, Fig. S1c, e). This construct, referred to as URAT1_EM_ hereafter, results in monomeric particles on cryo-EM grids (Supplementary information, Fig. S1b). Importantly, URAT1_EM_ shows urate transport activity with a K_M_ of 533.2 µM, similar to that reported for the WT transporter^30^ (Fig. 1a; Supplementary information, Fig. S1d). URAT1_EM_ also fully retains the sensitivity to anti-gout drugs lesinurad, verinurad, and dotinurad (Fig. 1b); in fact, it showed slightly higher sensitivity to lesinurad. This indicates that the modifications do not compromise the function and pharmacology of the transporter.

To understand the molecular basis of urate transport and drug inhibition, we determined the structures of URAT1_EM_ in the apo, substrate-bound (urate or counter anion pyrazinoate), and drug-bound states (Fig. 1c, d) with up to 2.7 Å resolution (Supplementary information, Figs. S2–S7 and Table S1). The density of the transporter is well-resolved, and the density of ligands is clearly visible, allowing reliable identification of the binding sites. URAT1 adopts a canonical major facilitator superfamily fold,^31,32^ with twelve transmembrane helices (TMs). TMs 1–6 form the N-domain, and TMs 7–12 form the C-domain (Fig. 1e, f). These two domains are linked by an intracellular helical domain (ICHD), composed of several short helices. Additionally, the loop between TM1 and TM2 constitutes an extracellular domain (Fig. 1e, f), a signature feature of the SLC22 family.^24,33^

In the apo state, the transporter is open to the intracellular side (inward-facing), with the central cavity between the N- and C-domains connected to the cytosol (Fig. 1g). The positive electrostatic potential of the cavity likely accounts for URAT1’s preference for anions (Supplementary information, Fig. S8a, b). By contrast, SLC22 family members OCT1 and OCT3, which prefer cations, maintain a negative electrostatic environment within their cavities^23–25^ (Supplementary information, Fig. S8c, d). In response to different ligands, URAT1 undergoes conformational changes that expose its cavity to different sides of the membrane for substrate transport (Fig. 1c), as we describe in detail below.

### Urate recognition

**Fig. 2:**
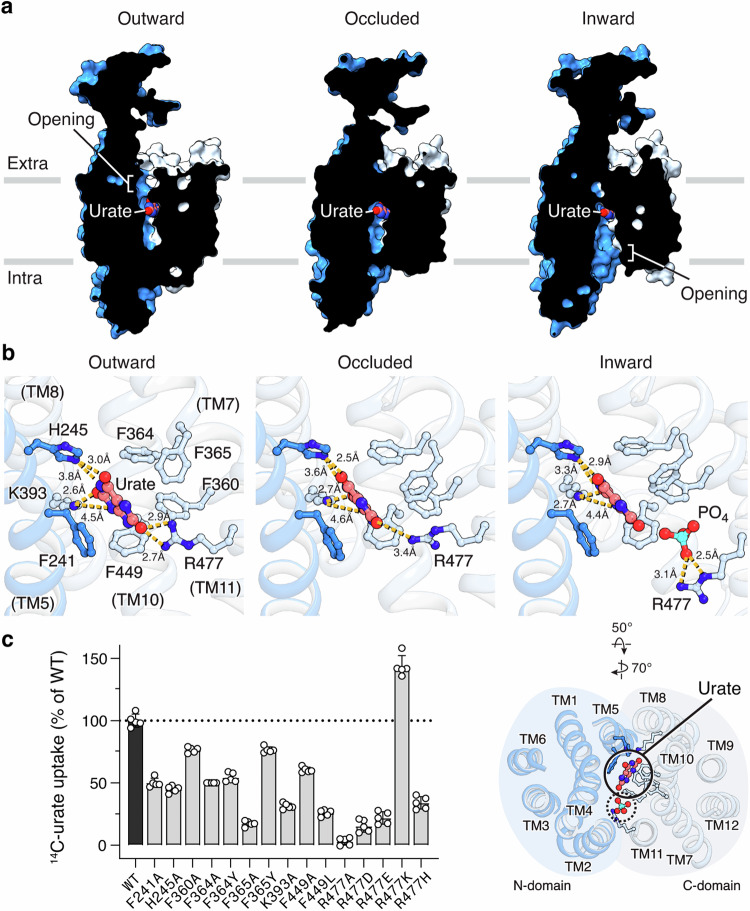
Urate recognition during the transport cycle of URAT1. **a** Slice view of URAT1 in the outward-facing, occluded, and inward-facing conformations, showing the urate pocket and the changes in access to the pocket. **b** Detailed interactions between urate and URAT1 in different conformations. **c** Urate uptake of URAT1 variants with single mutations in the urate-binding pocket, using the same buffer as defined in Fig. 1 legend. The graph shows individual data points, mean ± SD; *n* = 5 biological replicates.

In the presence of urate, we captured URAT1 in multiple conformations that represent major states in the transport cycle: outward-facing (cavity open to the extracellular side), occluded (cavity inaccessible to either side), and inward-facing (Fig. 2a; Supplementary information, Fig. S3). These structural snapshots reveal how URAT1 recognizes and imports urate. In all three conformations, urate binds to a similar location in the central cavity and this binding mode is notably different from previous predictions by molecular docking on homology models of URAT1.^34,35^ In our structures, the urate binding site is surrounded by several bulky aromatic residues, with five phenylalanines (Phe241, Phe360, Phe364, Phe365, and Phe449) forming a hydrophobic cage that encapsulates urate with hydrophobic and π–π stacking interactions (Fig. 2b; Supplementary information, Figs. S1e and S9a). Previous studies showed that mutation of Phe360, Phe364, or Phe365 reduces URAT1 activity.^35,36^ Here, we confirmed those observations and further found that mutating any of the five phenylalanines to alanine significantly reduced urate uptake (Fig. 2c), though the mutants could still traffic to the plasma membrane (Supplementary information, Fig. S10). These results support our structural findings that the phenylalanine cage residues directly participate in urate recognition. These residues are highly conserved across vertebrates,^37^ except for position 365, which is a tyrosine in rodents. Interestingly, rodent URAT1 has a lower affinity toward urate compared to its human ortholog,^37–39^ which could be attributed to the additional hydroxyl group on tyrosine causing steric clashes with urate (Supplementary information, Fig. S11). Indeed, we found that substituting tyrosine for either Phe365 or Phe364 in human URAT1 decreased urate uptake (Fig. 2c), indicating that those phenylalanine residues are important for high-affinity urate binding and that polymorphism in the phenylalanine cage may contribute to the differences in urate affinity across species.

In addition to the hydrophobic contacts described above, URAT1 also stabilizes urate binding through hydrophilic interactions. His245, Lys393, and Arg477 line the cavity and form hydrogen bonds with the nitrogen and hydroxyl atoms of urate (Fig. 2b). Mutations of those polar residues substantially reduced urate transport^34,35^ (Fig. 2c), highlighting the importance of these hydrogen bonds in urate uptake. Substituting alanine at residue 477 has the most drastic effect in our assays, as it completely abrogates urate uptake. We observed that as the transporter transitions from the outward-facing to the occluded conformations, the side chain of Arg477 swings away from the pocket, weakening its interaction with urate (Fig. 2b, left to middle). In the inward-facing conformation, Arg477 is completely flipped and its guanidinium group moves away and makes no interaction with urate (Fig. 2b, right). This gradual reduction in the Arg477 interaction with urate could facilitate release of the latter from the cavity into the cytoplasm.

There is a prominent density between urate and Arg477 in the inward-facing conformation. Because the buffer contains high concentrations of phosphate, we tentatively modeled this density as a phosphate molecule (Fig. 2b, right), but future experiments are required to further support this assignment. This anion site is approximately 5 Å apart from the putative chloride site in the related SLC22 transporter OAT1^21,40^ (Supplementary information, Fig. S12a). While we found that phosphate has minimal impact on URAT1 function (Supplementary information, Fig. S12b), as we demonstrated later, this site can also be bound by other anions that are transported by URAT1. Interactions between the anion and Arg477 likely stabilize the positively charged nitrogen atoms of the arginine, preventing it from re-engaging with the oxygen atom of urate. Supporting the importance of a positive residue at position 477 for URAT1 function, we found that R477D, R477E, and R477H (a variant associated with hypouricemia^10^) all hampered urate uptake, whereas R477K enhanced it (Fig. 2c).

These findings emphasize the crucial role of Arg477 in coupling the outward-inward transition of URAT1 with the coordination and transport of urate. This arginine residue is highly conserved in other SLC22 renal urate transporters, such as OAT1 and OAT4 (Supplementary information, Fig. S1e), suggesting that it participates in urate transport in those transporters as well.

### Conformational changes during urate transport

**Fig. 3:**
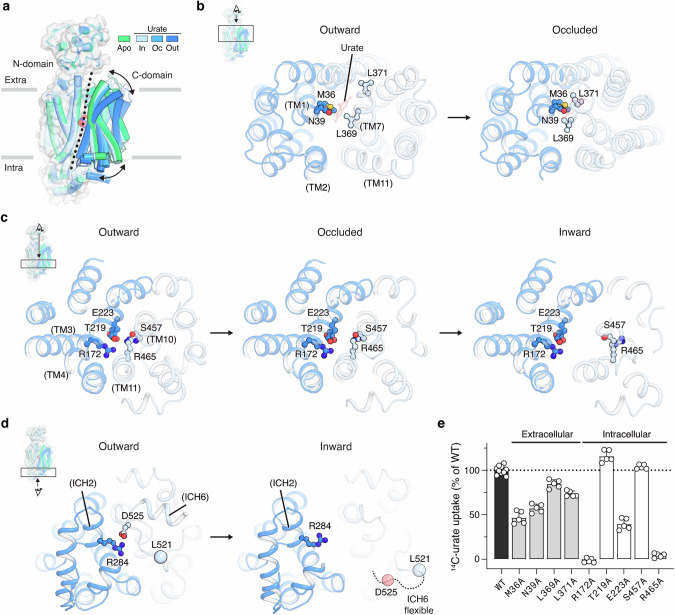
Conformational changes of URAT1 during urate transport. **a** Superposition of the apo and urate-bound conformations of URAT1. **b**–**d** Structural transitions in detail, view as indicated by boxes in insets. **b** The extracellular gate of URAT1, from outward-facing to occluded conformations. **c** The intracellular gate of URAT1, from outward-facing to occluded to inward-facing conformations. **d** The intracellular helical domain, from outward-facing to inward-facing conformations. ICH, intracellular helix. **e** Urate uptake of URAT1 variants with mutations in the extracellular and intracellular gates, using the same buffer as defined in Fig. 1 legend. The graph shows individual data points, mean ± SD; WT, *n* = 10 biological replicates; mutants, *n* = 5 biological replicates.

Superimposing the apo and urate-bound structures of URAT1 reveals the conformational transitions of the transporter during urate uptake (Fig. 3). The N- and C-domains move roughly as a rigid body, undergoing rocker-switch movements (Fig. 3a). Urate binding to the outward-facing conformation induces the closure of the extracellular gate in TM1 and TM7, leading to occlusion of the central cavity by Met36, Asn39, Leu369 and Leu371 (Fig. 3b; Supplementary information, Fig. S9b). Mutations of any of these residues to alanine reduced urate uptake, with a more pronounced effect when substituting the larger ones, Met36 and Asn39 (Fig. 3e).

The transitions from outward-facing to occluded to inward-facing conformations also include the opening of the intracellular gate. This gate is composed of several polar and charged residues, such as Arg172, Glu223, and Arg465, on the cytoplasmic side of TMs 3, 4, 10, and 11. In the outward-facing conformation, hydrophilic interactions between those residues seal the gate (Fig. 3c; Supplementary information, Fig. S9c), which is further stapled by the ionic interaction between Arg284 of the intracellular helix 2 and Asp525 near the intracellular helix 6 (Fig. 3d). The intracellular gate begins to loosen in the occluded state and fully opens in the inward-facing state, permitting urate release into the cell (Fig. 3c). The rocker-switch movements also disrupt the interactions between intracellular helices 2 and 6 (Supplementary information, Fig. S9d). In the inward-facing conformation, intracellular helix 6 becomes highly flexible, which renders the residues beyond Leu521 invisible in the cryo-EM map (Fig. 3d). Mutating the charged residues in the intracellular gate to alanine disrupted urate uptake (Fig. 3e), supporting their role in the transport cycle. Together, our structural and functional data pinpoint critical residues in the transporter gates and elucidate how urate-dependent gating occurs.

### Binding of the intracellular counter anion

**Fig. 4:**
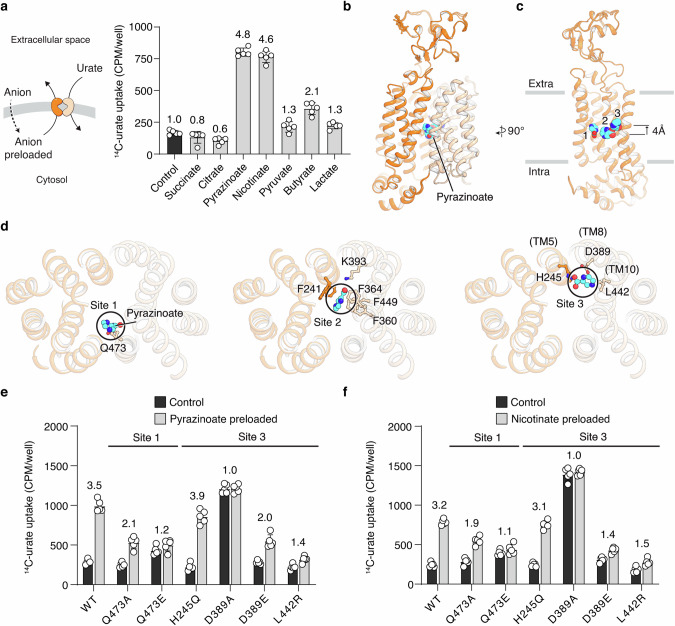
Recognition of counter anion pyrazinoate by URAT1. **a** Urate uptake of cells preloaded with various organic anions. The potentiation in urate uptake by anion preloading is presented as fold changes. Uptake buffer: 20 mM HEPES pH 7.4, 125 mM sodium chloride, and 5.6 mM glucose. Graph shows individual data points, mean ± SD; *n* = 5 biological replicates. **b** Pyrazinoate-bound URAT1 in an inward-facing conformation. **c** Three different pyrazinoate-binding sites in URAT1. For clarity, only the N-domain is shown. **d** Detailed interactions between pyrazinoate and URAT1, depicted in a top view looking down from the extracellular side. **e** Pyrazinoate potentiation of urate uptake. URAT1 variants with mutations in Sites 1 and 3 were examined. The potentiation in urate uptake by pyrazinoate preloading is presented as fold changes. Same buffer as in **a**. The graph shows individual data points, mean ± SD; *n* = 5 biological replicates. **f** Nicotinate potentiation on urate uptake. URAT1 variants with mutations in Sites 1 and 3 were examined. The potentiation in urate uptake by nicotinate preloading is presented as fold changes. The graph shows individual data points, mean ± SD; *n* = 5 biological replicates; same buffer as in **a**.

URAT1 is an antiporter, exporting intracellular organic anions, such as nicotinate or pyrazinoate, while transporting extracellular urate into cells. We preloaded cells with pyrazinoate and nicotinate and observed a substantial increase in urate uptake (Fig. 4a), confirming previous reports that URAT1 can use these anions to drive urate transport.^6,14^ We also found that butyrate could act as a counter anion for URAT1 (Fig. 4a), although less effectively.

Due to its strong effect, we used pyrazinoate to explore the structural basis of intracellular counter anion transport and determined the structures of URAT1 with pyrazinoate. The transporter was observed in an inward-facing conformation (Fig. 4b) and through 3D classification (Supplementary information, Fig. S4), we identified three distinct populations, each harboring a pyrazinoate molecule bound in a different site within URAT1, which we designate as Sites 1, 2, and 3. The three structures are globally similar, with rearrangements occurring locally at the pyrazinoate binding sites (Supplementary information, Fig. S13a). Sites 1 and 2 are located approximately halfway through the membrane, whereas Site 3 is 4 Å above Site 2 and closer to the extracellular side (Fig. 4c). Pyrazinoate in Site 1 interacts with Gln473 (Fig. 4d, left; Supplementary information, Fig. S13b), and it overlaps with the position of the phosphate molecule in the urate-bound inward-facing structure (Supplementary information, Fig. S13e), providing additional evidence that this location is an anion binding site. Site 2 is lodged in the central cavity and overlaps with the urate site, with pyrazinoate interacting with a set of residues similar to those interacting with urates, such as the phenylalanine cage and Lys393 (Fig. 4d, middle; Supplementary information, Fig. S13c). Site 3 is surrounded by His245, Asp389, and Leu442 of TM5, TM8, and TM10, respectively (Fig. 4d, right; Supplementary information, Fig. S13d).

We validated these structural observations by mutagenesis. Mutations in Site 2 compromised urate transport and reduced the signal-to-noise ratio, complicating the interpretation; thus, we focused on Site 1 and Site 3. Mutations in Site 1 reduced the urate uptake potentiation effect by anion preloading from 3.5-fold to 1.2-fold (Fig. 4e). Mutations in Site 3 also reduced or even completely abolished the potentiation (Fig. 4e). Considering that pyrazinoate is an exogenous metabolite, we also examined the impact of these mutations on the potentiation mediated by the endogenous metabolite nicotinate (Fig. 4f). The effects observed were similar to those with pyrazinoate, suggesting that URAT1 uses the same sites to transport nicotinate and other endogenous organic anions.

Site 3 in URAT1 appears to be reminiscent of the counter anion site in OAT1, which binds α-ketoglutarate in a pocket formed by TM5, TM8, and TM10.^21^ However, URAT1 Site 3 is close to the extracellular side, barely overlapping with the α-ketoglutarate site in OAT1 (Supplementary information, Fig. S13f). The different chemical environment of the binding pockets of these two transporters may dictate their substrate selectivity: URAT1 transports pyrazinoate but not α-ketoglutarate, whereas OAT1 transports α-ketoglutarate but is inhibited by pyrazinoate.^41^ In OAT1, α-ketoglutarate binding was proposed to break a salt bridge involving Lys431 to initiate a structural transition. While this lysine is conserved in other SLC22 renal urate transporters (Supplementary information, Fig. S1e), URAT1 features a leucine at this position that is incapable of forming any hydrophilic interactions, indicating that different gating machinery must have evolved in URAT1. Furthermore, in contrast to OAT1, which has only one single α-ketoglutarate site,^21^ URAT1 possesses three individual sites for the counter anion. These observations suggest that URAT1 employs a unique mechanism to recognize and export intracellular anions.

### Mechanism of URAT1 inhibition by lesinured and verinurad

**Fig. 5:**
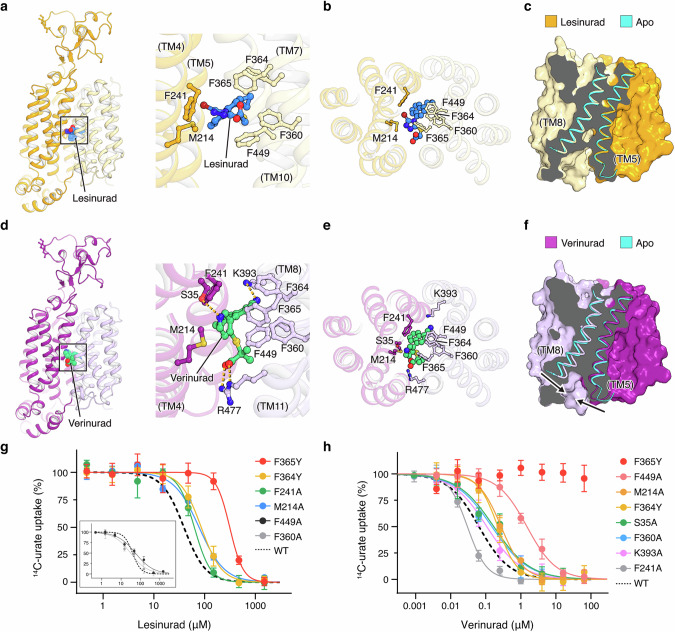
Mechanism of URAT1 inhibition by lesinurad and verinurad. **a** Structure of lesinurad-bound URAT1. Detailed interactions between lesinurad and URAT1 are shown on the right. **b** Lesinurad-binding pocket, depicted in a top view looking down from the extracellular side. **c** Comparison between the lesinurad-bound and apo structures. **d** Structure of verinurad-bound URAT1. Detailed interactions between verinurad and URAT1 are shown on the right. **e** Verinurad-binding pocket, depicted in a top view looking down from the extracellular side. **f** Comparison between the verinurad-bound and apo structures. **g** Dose-response curves of URAT1 variants with mutations in the lesinurad-binding site. For clarity, the data of F449A and F360A are shown in the inset. Data are shown as mean ± SD; *n* = 3 biological replicates. **h** Dose-response curves of URAT1 variants with mutations in the verinurad-binding site. Data are shown as mean ± SD; *n* = 3 biological replicates. In **g**, **h**, uptake buffer used was the same as defined in Fig. 1 legend.

To understand how anti-gout drugs inhibit URAT1, we determined the structures of URAT1 bound to lesinurad or verinurad (Fig. 5). URAT1 adopts an inward-facing conformation when bound to lesinurad, with the drug occupying the central cavity (Fig. 5a). Lesinurad is stabilized by hydrophobic and π–π interactions from multiple aromatic residues and Met214, with its naphthalene moiety in the center of the phenylalanine cage (Fig. 5a, b). This binding pose largely overlaps with those of urate and pyrazinoate at Site 2, indicating that lesinurad directly competes with substrates to inhibit URAT1. Mutations in the residues lining the lesinurad binding pocket reduced drug potency (Fig. 5g), with the most pronounced reduction observed with F365Y (IC_50_ = 312.0 µM, compared to 39.0 µM of the WT URAT1). The additional hydroxyl group on tyrosine likely clashes with lesinurad, similar to the impact of F365Y on urate binding. Consistent with this idea, rodent URAT1, which bears a tyrosine at this position, has a much lower affinity for lesinurad.^36^ Globally, the structure of lesinurad-bound URAT1 resembles the apo state (Fig. 5c), with the intracellular vestibule more open than that in the urate-bound, inward-facing conformation (Fig. 3a). Thus, in addition to directly competing with the substrates, lesinurad binding likely traps URAT1 in an apo-like conformation and arrests the transport cycle of urate.

A more potent derivative of lesinurad (Fig. 1b), verinurad,^20^ shares a similar naphthalene backbone. Our structure of URAT1 in complex with verinurad shows that this drug also binds in the central cavity (Fig. 5d), but forms considerably more hydrophilic interactions with URAT1 compared to lesinurad (Fig. 5d, e). Specifically, nitrogen atoms on the pyridine ring and the cyano group of lesinurad form hydrogen bonds with URAT1 Ser35 and Lys393, respectively, and the carboxyl group interacts with Arg477, an important gating residue (Fig. 5d, e). This extensive interaction network securely positions verinurad within URAT1, preventing substrates from accessing the central cavity. Consistently, mutating the involved residues perturbed verinurad’s action, with F365Y rendering the transporter insensitive to the drug (Fig. 5h). Interestingly, F365Y and F449A have much stronger effects on verinurad than on lesinurad, demonstrating that while both drugs interact with some common residues, specific interactions due to subtle differences in the drug geometry could lead to profoundly different drug effects. Furthermore, in contrast to the apo-like conformation induced by lesinurad, verinurad binding and engagement of Arg477 are accompanied by a reconfiguration of the intracellular vestibule, including TM5 and TM8 (Fig. 5f), which leads to a narrower opening toward the cytoplasm. Thus, the two compounds stabilize the transporters in different conformations, which could also contribute to their different pharmacology.

### Mechanism of URAT1 inhibition by dotinurad

**Fig. 6:**
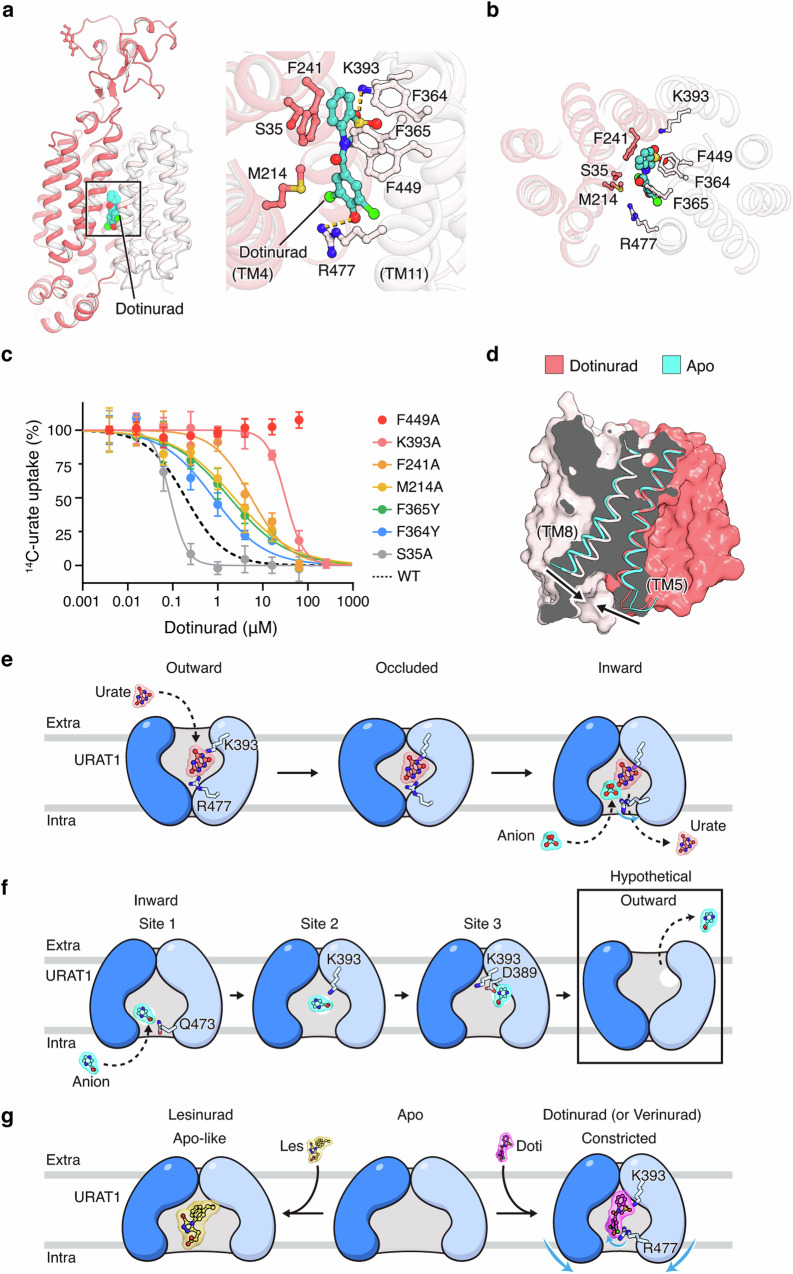
Dotinurad inhibition and the mechanism of URAT1 transport. **a** Structure of dotinurad-bound URAT1. Detailed interactions between dotinurad and URAT1 are shown on the right. **b** Dotinurad-binding pocket, depicted in a top view looking down from the extracellular side. **c** Dose-response curves of URAT1 variants with mutations in the dotinurad-binding site, using the same buffer as defined in Fig. 1 legend. Data are shown as mean ± SD; *n* = 3 biological replicates. **d** Comparison between the dotinurad-bound and apo structures. **e** Schematic diagram of URAT1 importing urate. The N-domain and C-domain are shown in different shades of blue. **f** Schematic diagram of URAT1 exporting the intracellular counter anion. The conformation in the box has yet to be captured experimentally. It is possible that an anion directly enters Site 2, but for simplicity this pathway is not depicted here. **g** Two modes of drug inhibition: High-affinity drugs verinurad and dotinurad engage Lys393 and Arg477, and induce additional conformational changes, compared to lesinurad.

To further elucidate the mechanism of inhibitors targeting URAT1, we sought to investigate compounds with distinct chemotypes. Dotinurad contains a benzothiazole moiety instead of the naphthalene in lesinurad and verinurad.^19^ We determined the structure of dotinurad-bound URAT1 (Fig. 6a) and found that the benzothiazole core of dotinurad occupies the phenylalanine cage, resting above Phe449 and forming a π–π stacking interaction with Phe241 (Fig. 6a, b). Notably, dotinurad’s hydroxyl groups form hydrogen bonds with Lys393 and Arg477, two critical residues that couple urate/counter-anion exchange with transporter conformational changes. These interactions are reminiscent of versinurad (Fig. 5d, e), indicating that these two high-potency drugs inhibit URAT1 by exploiting a common mechanistic hotspot central to the transport cycle.

Mutating URAT1 residues lining the drug pocket reduced dotinurad’s potency (Fig. 6c), with F449A completely eliminating URAT1’s sensitivity to dotinurad. F365Y had a milder influence on dotinurad’s potency (Fig. 6c), in contrast to the drastic effect seen with lesinurad and verinurad (Fig. 5g, h). This observation is consistent with the structural data, showing that dotinurad is positioned in the phenylalanine cage at an angle that diverges from that of naphthalene-based compounds, which would allow it to better tolerate the additional hydroxyl group in F365Y. Dotinurad locks the transporter in an inward-facing conformation, with the cytoplasmic part of TM5 and TM8 bent toward each other and constricting the intracellular opening (Fig. 6d). These conformational changes resemble those induced by verinurad binding, highlighting that locking URAT1 in this constricted conformation is a common and effective strategy for potent inhibition by various drugs.

## Discussion

Our structures of human URAT1 in complex with substrates urate and pyrazinoate provide mechanistic insights into how the transporter takes up extracellular urate while exporting intracellular anions. Based on our structural and functional analyses, we propose the following transport mechanism of URAT1. When the transporter is in its outward-facing conformation, extracellular urate enters the phenylalanine-rich central binding pocket and interacts with gating residues Lys393 and Arg477 (Fig. 6e). These interactions drive conformational changes that close the extracellular gate, followed by the expansion of the intracellular gate. In this inward-facing conformation, intracellular anions can access the transporter at Site 1 and disrupt the interaction between urate and Arg477, pushing the Arg477 side chain away from the pocket and allowing urate to be released into the cytosol. Electrostatic repulsion between the urate and the counter anion in the pocket may also facilitate the process.

Once urate is released, transportable anions such as pyrazinoate migrate to the central cavity (Site 2) and eventually to Site 3 (Fig. 6f), which is closer to the extracellular side and primed for export. Such a sequential model for counter-anion efflux contrasts with the mechanisms proposed for other anion antiporters, suggesting a new mode of organic anion transport. We observed that when pyrazinoate binds to Site 3, the side chains of Asp389 and Lys393 appear to rearrange, compared to the apo state (Supplementary information, Fig. S13g). Although the side chain density of Asp389 is less well-resolved, we hypothesize that anion binding to Site 3 likely causes the γ-carboxylate of Asp389 to move toward Lys393, thereby altering their interaction. We speculate that the displacement of Lys393 on TM8 may reduce its interaction with the neighboring TM5, allowing the cytoplasmic halves of TM8 and TM5 to coalesce, which eventually triggers the inward-to-outward transition of URAT1. Consistent with this proposal, Lys393 is important for URAT1 activity (Fig. 2c) and the corresponding residue in OAT1 (Lys382) has been proposed to be involved in conformational switches.^21^

The mechanism of urate recognition in URAT1 is drastically different from that of another urate transporter, GLUT9 (SLC2A9).^42^ GLUT9 does not have the phenylalanine cage present in URAT1, nor does it use charged residues to coordinate urate (Supplementary information, Fig. S14). Since Arg477 and Lys393 in URAT1 couple urate binding with transporter conformational changes, the absence of these charged residues in GLUT9 suggests that it likely employs a different mechanism to transport urate.

Our structures of human URAT1 provide a better understanding for how certain URAT1 mutations found in patients may lead to hypouricemia^6,9–12,43–48^ (Supplementary information, Fig. S15). For example, Arg477 directly binds urate; the R477H mutation in patients would interfere with that interaction, resulting in reduced urate transport. Gly366 is located right next to the phenylalanine cage that forms the urate pocket; the G366R mutation in patients potentially rigidifies the transmembrane helix, which can perturb the cage and reduce urate transport; moreover, the introduction of a large, charged side chain at this position may affect URAT1 folding and trafficking.

Our structures of URAT1 with three anti-gout drugs — leinurad, verinurad and dotinurad — reveal how they exploit URAT1’s key gating residues to potently inhibit transport activity. All three drugs occupy the phenylalanine cage, thus competing with URAT1 substrates. In addition, the drugs trap the transporter in inward-facing conformations, halting the transport cycle. The low-affinity inhibitor lesinurad stabilizes the transporter in an apo-like conformation, whereas the high-affinity inhibitors verinurad and dotinurad stabilize the transporter in a constricted open state (Fig. 6g). Furthermore, verinurad and dotinurad both engage with Arg477, an interaction correlated with the bending of TM5 and TM8 and narrowing of the intracellular opening. Engaging Arg477 can be explored and incorporated into the design of future compounds targeting URAT1. Our work thus provides a foundation for the rational development of future anti-gout therapeutics.

## Materials and methods

### ^14^C-Urate uptake assays

Human embryonic kidney (HEK) 293 cells (ATCC, CRL-3216) were seeded onto poly(D-lysine)-coated 96-well plates at a density of 0.3 × 10^5^ cells per well and cultured at 37 °C until reaching approximately 70%–80% confluence. The cells were transiently transfected with plasmids encoding the *URAT1* WT or mutants using Lipofectamine 3000 (Invitrogen, L3000001), following the manufacturer’s protocol. The transfected cells were cultured at 37 °C for 12 h. Following this, 10 mM sodium butyrate was added, and the cells were further incubated at 30 °C for an additional 24 h. The medium was aspirated, and the cells were rinsed once with assay buffer (20 mM HEPES pH 7.4, 125 mM sodium gluconate, 4.8 mM potassium gluconate, 1.2 mM monobasic potassium phosphate, 1.2 mM magnesium sulfate, 1.3 mM calcium gluconate, and 5.6 mM glucose) and then incubated with 400 µM urate consisted of 80 µM ^14^C-radiolabeled (50–60 mCi/mmol, 0.5 mCi/mL, American Radiolabeled Chemicals, Inc.) and 320 µM non-radiolabeled in assay buffer at 37 °C for 20 min. In some experiments, 125 mM sodium gluconate was replaced by sodium chloride, sulfate, or phosphate. Uptake was stopped by removing the substrate-containing buffer and washing the cells twice with ice-cold Dulbecco’s phosphate-buffered saline (Gibco, 14190144). The cells were then lysed in 0.1 M NaOH, and the lysed samples were added to scintillation liquid to measure radioactivity using a PerkinElmer Microbeta2 counter.

For dose-response assays, the cells were incubated with urate and increasing concentrations of inhibitors. For anion pre-oading assays, sodium butyrate was omitted, and anions were preloaded by incubation with cells for 2 h in Dulbecco’s modified Eagle’s medium at 37 °C. The medium was then aspirated, and urate uptake was initiated by incubating cells in 400 µM urate (80 µM ^14^C-radiolabeled and 320 µM non-radiolabeled) in pre-warmed uptake buffer (20 mM HEPES pH 7.4, 125 mM sodium chloride, and 5.6 mM glucose) for 20 min at 37 °C. Nonspecific uptake was measured in each plate in non-transfected cells, and this value was subtracted from the total uptake to obtain the URAT1-specific uptake. To compare the uptake signals among URAT1 variants, the mVenus fluorescence intensity of each construct was measured using fluorescence-detection size-exclusion chromatography^49^ (λ_ex_/λ_em_: 510 nm/530 nm). The intensity of each variant was divided by that of the WT transporter to determine the differences in expression levels. These ratios were subsequently applied to normalize the uptake signal for each variant. IC_50_ values of drugs were determined by fitting the data to three-parameter or four-parameter dose-response curves using GraphPad Prism 9.

### Expression and purification of URAT1 for cryo-EM

The complementary DNA encoding human URAT1 was cloned into a modified pEG BacMam vector.^50^ For URAT1_EM_, an N-terminal mVenus tag^51^ and a 3C protease cleavage site were inserted before the coding sequence of MBP, followed by a helical linker, human URAT1 (residues 3–553), a (GGGGS)3 linker, and C-terminal DARPin_off7_ (Supplementary information, Fig. S1c). An intracellular region (residues 280–343) and an extracellular loop (residues 60–65) were replaced with the corresponding sequences from human OAT4 (Supplementary information, Fig. S1e). URAT1_EM_ was expressed in HEK293S GnTI^−^ cells (ATCC, CRL-3022) using the BacMam system. Baculoviruses were produced by transfecting Sf9 cells (ATCC, CRL-1711) with bacmids using Mirus TransIT-Insect reagents (Mirus Bio, MIR 6100). After one or two rounds of amplification, viruses were used for cell transduction. When HEK293S GnTI^−^ suspension cultures reached a density of 5 × 10^6^ cells per mL after growth at 37 °C, baculoviruses (10% (v/v)) were added to initiate transduction. After 16–18 h, the cultures were supplemented with 10 mM sodium butyrate, and the culture temperature was shifted to 25 °C. Cells were collected at 60 h after transduction and stored at −80 °C.

Frozen cell pellets were thawed at room temperature and then resuspended in hypotonic buffer (20 mM MES pH 6.5, 10 mM NaCl, 1 mM MgCl_2_, protease inhibitor cocktail and benzonase, and 0.1 mM TCEP) for 20 min on ice. The cell lysate was then spun at 39,800× *g* for 30 min to sediment crude membranes. The membrane pellet was mechanically homogenized in MBS buffer (20 mM MES pH 6.5, 300 mM NaCl, 0.1 mM TCEP, and protease inhibitor cocktail). The suspension was solubilized in 1.2% (w/v) digitonin for 90 min at 4 °C. The solubilized material was centrifuged at 39,800× *g* for 45 min, and the supernatant was incubated with anti-GFP nanobody resins for 2 h at 4 °C. Resins were then washed with 20 column volumes of wash buffer A (40 mM MES pH 6.5, 0.05% digitonin, 150 mM NaCl, 150 mM KCl, 4 mM MgCl_2_, 4 mM NaATP, and 0.1 mM TCEP) followed by 10 column volumes of wash buffer B (20 mM MES pH 6.5, 0.05% digitonin, 150 mM NaCl, and 0.1 mM TCEP). The washed resin was incubated with saposin A^52^ at a URAT1 to saposin ratio of 1:20 (molar ratio). After 30 min, γ-cyclodextrin was added to the resin to remove digitonin and facilitate nanoparticle reconstitution overnight. The resin was then washed with 15 column volumes of wash buffer C (20 mM MES pH 6.5, 150 mM NaCl, and 0.1 mM TCEP). The washed resin was incubated with 3C protease for 2 h at a target protein-to-protease ratio of 40:1 (w/w) to cleave off mVenus and release the protein from the resin. The protein was eluted with wash buffer C, concentrated, and further purified by gel-filtration chromatography on a Superose 6 increase column equilibrated with wash buffer C. The peak fractions of protein were pooled and concentrated to ~8.0 mg/mL.

### Cryo-EM sample preparation and data acquisition

For lesinurad-bound, verinurad-bound, and dotinurad-bound complexes, stocks of the compounds dissolved in DMSO were added to the protein sample at final concentrations of 1.6 mM, 0.75 mM, and 1.5 mM, respectively. For urate-bound or pyrazinoate-bound complexes, solutions of urate (15 mM stock in 50 mM Na_2_HPO_4_, 15 mM NaOH, final pH ~8.5) or pyrazinoate (18 mM stock in 50 mM Tris, 20 mM NaOH, final pH ~8.5) was added to the sample at final concentrations of 7.5 mM and 9 mM, respectively. For all cryo-EM experiments, a 3.5-μL volume of sample supplemented with 0.4 mM fluorinated octyl maltoside was applied to plasma-cleaned UltrAuFoil R1.2/1.3 300-mesh grids (Quantifoil) under 100% humidity at 10 °C. The grids were blotted for 3.0 s and plunged into liquid ethane using a Vitrobot Mark IV (ThermoFisher Scientific), and subsequently imaged at cryogenic temperatures under a 300 kV Titan Krios G3 transmission electron microscope equipped with a post-column energy filter using EPU software. Raw movie stacks were recorded with a K3 camera at a physical pixel size of 0.649 Å per pixel and a nominal defocus range of 1.1–2.1 µm. The exposure time for each micrograph was 1.8–2.0 s, fractionated into 60–70 frames with a dose rate of 0.98–1.21 e^–^ per Å^2^ per second. The data collection parameters are summarized in Supplementary information, Table S1.

### Cryo-EM image processing

The data processing flowcharts are shown in Supplementary information, Figs. S2–S7. Briefly, the image stacks were gain-normalized and corrected for beam-induced motion using MotionCor2.^53^ Defocus parameters were estimated from motion-corrected images using cryoSPARC4.^54^ Micrographs not suitable for further analysis were removed by manual inspections. Particle pickings (blob and template picker) and two-dimensional (2D) classifications were done in cryoSPARC4 (Supplementary information, Figs. S2a, S3a, S4a, S5a, S6a, and S7a). After 2D classification, selected particles were used for training in the Topaz particle-picking pipeline.^55^ Particles picked by Topaz were subjected to 2D classification. Selected particles were combined with particles from blob picker and template picker, and duplicated particles were removed. Iterative three-dimensional (3D) classifications were then performed with subsequent ab initio reconstructions and heterogeneous refinements to remove suboptimal particles. Selected particles were refined using non-uniform refinement,^56^ followed by local refinements with soft masks covering URAT1 to further improve map quality. The refined particles were subjected to Bayesian polishing in RELION4.^57^ The polished particles were imported into cryoSPARC4, where additional heterogeneous, non-uniform, local, and CTF refinements were performed. For the pyrazinoate and urate bound datasets, additional 3D classifications were performed before the final non-uniform and local refinement in cryoSPARC4 (Supplementary information, Figs. S3, S4). Mask-corrected FSC curves were calculated in cryoSPARC4 (Supplementary information, Figs. S2b, S3b–d, S4b–d, S5b, S6b, and S7b), and reported resolutions are based on the 0.143 criterion. Local resolution estimations were performed in cryoSPARC4 (Supplementary information, Figs. S2d, S3h–j, S4h–j, S5d, S6d, and S7d).

### Model building and refinement

An initial model of human URAT1 was generated by AlphaFold.^58^ This model was docked into the density maps using Chimera.^59^ The model was then refined iteratively using Coot,^60^ ISOLDE,^61^ and Phenix.^62^ Structural model validation was performed using Phenix and MolProbity (Supplementary information, Table S1).^63^ Figures were prepared using PyMOL, Chimera, and ChimeraX.

### Fluorescence microscopy

To evaluate the cellular localization of URAT1 mutants, HEK293 cells were co-transfected with constructs of mVenus-tagged URAT1 and mCherry-tagged Spns2 (Supplementary information, Fig. S10). A day prior to transfection, HEK293 cells were plated onto poly(D-lysine)-coated 8-well glass chamber coverglasses at a density of 0.15 × 10^5^ cells per well. Transfection was carried out using Promega FuGENE HD, following the manufacturer’s protocol. After transfection, cells were incubated at 37 °C for 36 h, subsequently rinsed with PBS, and fixed with 4% paraformaldehyde for 20 min. Following fixation, cells were washed twice with PBS. Imaging was performed on a Zeiss LSM 780 microscope using a 40× objective lens, with mVenus fluorescence excited at 498 nm and detected at 553 nm, and mCherry fluorescence excited at 578 nm and detected at 695 nm. Image analysis was performed using ImageJ software.

## Supplementary information

Supplementary information Fig S1

Supplementary information Fig S2

Supplementary information Fig S3

Supplementary information Fig S4

Supplementary information Fig S5

Supplementary information Fig S6

Supplementary information Fig S7

Supplementary information Fig S8

Supplementary information Fig S9

Supplementary information Fig S10

Supplementary information Fig S11

Supplementary information Fig S12

Supplementary information Fig S13

Supplementary information Fig S14

Supplementary information Fig S15

Supplementary information Table S1

## Data Availability

Cryo-EM maps and coordinates have been deposited in the EMDB and wwPDB, respectively, with accession numbers: EMD-44077 and PDB 9B1F (apo); EMD-44083 and PDB 9B1L (urate-bound, outward-facing); EMD-44082 and PDB 9B1K (urate-bound, occluded state); EMD-44081 and PDB 9B1J (urate-bound, inward-facing); EMD-44084 and PDB 9B1M (pyrazinoate-bound, Site 1); EMD-44085 and PDB 9B1N (pyrazinoate-bound, Site 2); EMD-44086 and PDB 9B1O (pyrazinoate-bound, Site 3); EMD-44079 and PDB 9B1H (lesinurad-bound); EMD-44080 and PDB 9B1I (verinurad-bound); EMD-44078 and PDB 9B1G (dotinurad-bound).
