# Supplementary material for: Transport mechanism and structural pharmacology of human urate transporter URAT1
Source: Cell Res. 2024 Sep 9;34(11):776–87. doi: 10.1038/s41422-024-01023-1 (PMC11528023; doi:10.1038/s41422-024-01023-1)
Supplement: Supplementary file 1 — Supplementary information Fig S1 [file 41422_2024_1023_MOESM1_ESM.pdf]

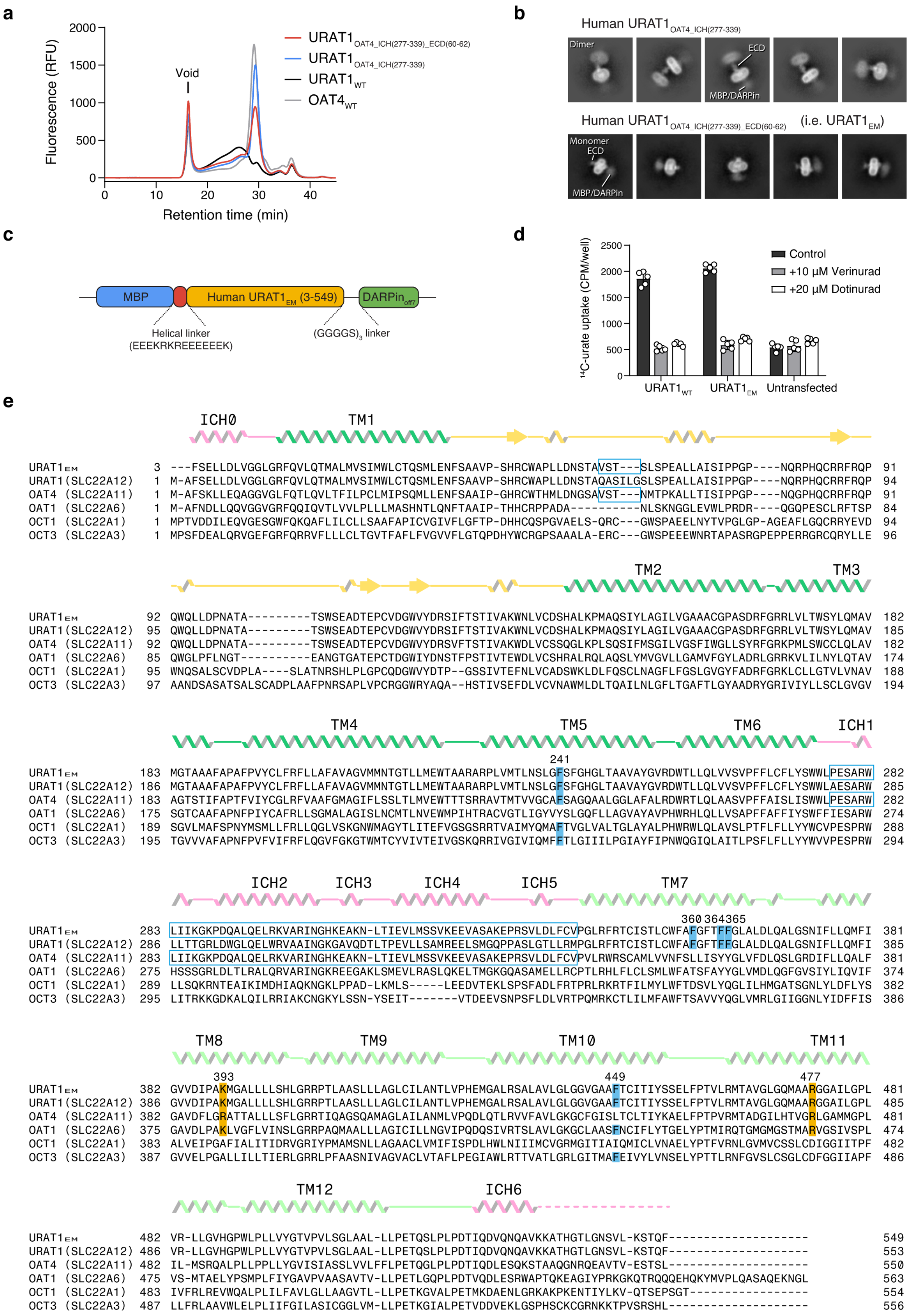
a
2000
1500
1000
500
0
Fluorescence (RFU)
Void
0
10
20
30
40
Retention time (min)
URAT1 OAT4_ICH(277-339)_ECD(60-62)
URAT1 OAT4_ICH(277-339)
URAT1 WT
OAT4 WT
b
Human URAT1 OAT4_ICH(277-339)
Dimer
ECD
MBP/DARPin
Human URAT1 OAT4_ICH(277-339)_ECD(60-62) (i.e. URAT1 EM)
Monomer
ECD
MBP/DARPin
c
MBP
Human URAT1 EM (3-549)
DARPin off7
Helical linker
(EEEKRKREEEEEEK)
(GGGGS)3 linker
d
2500
2000
1500
1000
500
0
14C-urate uptake (CPM/well)
Control
+10 μM Verinurad
+20 μM Dotinurad
URAT1 WT
URAT1 EM
Untransfected
e
ICH0
TM1
URAT1EM 3 ---FSELLDLVGGLGRFQVLQTMALMVSIMWLCTQSMLENFSAAVP-SHRCWAPLLDNSTAVST---SLSPEALLAISIPPGP----NQRPHQCRRFRQP 91
URAT1(SLC22A12) 1 M-AFSELLDLVGGLGRFQVLQTMALMVSIMWLCTQSMLENFSAAVP-SHRCWAPLLDNSTAQASILGSLSPEALLAISIPPGP----NQRPHQCRRFRQP 94
OAT4 (SLC22A11) 1 M-AFSKLLEQAGGVGLFQTLQVLTFILPCLMIPSQMLLENFSAAIP-GHRCWTHMLDNGSAVST---NMTPKALLTISIPPGP----NQGPHQCRRFRQP 91
OAT1 (SLC22A6) 1 M-AFNDLLQQVGGVGRFQQIQVTLVVLPLLLMASHNTLQNFTAAIP-THHCRPPADA----------NLSKNGGLEVWLPRDR----QGQPESCLRFTSP 84
OCT1 (SLC22A1) 1 MPTVDDILEQVGESGWFQKQAFLILCLLSAAFAPICVGIVFLGFTP-DHHCQSPGVAELS-QRC---GWSPAEELNYTVPGLGP-AGEAFLGQCRRYEVD 94
OCT3 (SLC22A3) 1 MPSFDEALQRVGEFGRFQRRVFLLLCLTGVTFAFLFVGVVFLGTQPDHYWCRGPSAAALA-ERC---GWSPEEEWNRTAPASRGPEPPERRGRCQRYLLE 96
TM2
TM3
URAT1EM 92 QWQLLDPNATA---------TSWSEADTEPCVDGWVYDRSIFTSTIVAKWNLVCDSHALKPMAQSIYLAGILVGAAACGPASDRFGRRLVLTWSYLQMAV 182
URAT1(SLC22A12) 95 QWQLLDPNATA---------TSWSEADTEPCVDGWVYDRSIFTSTIVAKWNLVCDSHALKPMAQSIYLAGILVGAAACGPASDRFGRRLVLTWSYLQMAV 185
OAT4 (SLC22A11) 92 QWQLLDPNATA---------TSWSEADTEPCVDGWVYDRSVFTSTIVAKWDLVCSSQGLKPLSQSIFMSGILVGSFIWGLLSYRFGRKPMLSWCCLQLAV 182
OAT1 (SLC22A6) 85 QWGLPFLNGT----------EANGTGATEPCTDGWIYDNSTFPSTIVTEWDLVCSHRALRQLAQSLYMVGVLLGAMVFGYLADRLGRRKVLILNYLQTAV 174
OCT1 (SLC22A1) 95 WNQSALSCVDPLA----SLATNRSHLPLGPCQDGWVYDTP--GSSIVTEFNLVCADSWKLDLFQSCLNAGFLFGSLGVGYFADRFGRKLCLLGTVLVNAV 188
OCT3 (SLC22A3) 97 AANDSASATSALSCADPLAAFPNRSAPLVPCRGGWRYAQA--HSTIVSEFDLVCVNAWMLDLTQAILNLGFLTGAFTLGYAADRYGRIVIYLLSCLGVGV 194
TM4
TM5
TM6
ICH1
241
URAT1EM 183 MGTAAAFAPAFPVYCLFRFLLAFAVAGVMMNTGTLLMEWTAARARPLVMTLNSLGFSFGHGLTAAVAYGVRDWTLLQLVVSVPFFLCFLYSWWLPESARW 282
URAT1(SLC22A12) 186 MGTAAAFAPAFPVYCLFRFLLAFAVAGVMMNTGTLLMEWTAARARPLVMTLNSLGFSFGHGLTAAVAYGVRDWTLLQLVVSVPFFLCFLYSWWLAESARW 285
OAT4 (SLC22A11) 183 AGTSTIFAPTFVIYCGLRFVAAFGMAGIFLSSLTLMVEWTTTSRRAVTMTVVGCAFSAGQAALGGLAFALRDWRTLQLAASVPFFAISLISWWLPESARW 282
OAT1 (SLC22A6) 175 SGTCAAFAPNFPIYCAFRLLSGMALAGISLNCMTLNVEWMPIHTRACVGTLIGYVYSLGQFLLAGVAYAVPHWRHLQLLVSAPFFAFFIYSWFFIESARW 274
OCT1 (SLC22A1) 189 SGVLMAFSPNYMSMLLFRLLQGLVSKGNWMAGYTLITEFVGSGSRRTVAIMYQMAFTVGLVALTGLAYALPHWRWLQLAVSLPTFLFLLYYWCVPESPRW 288
OCT3 (SLC22A3) 195 TGVVVAFAPNFPVFVIFRFLQGVFGKGTWMTCYVIVTEIVGSKQRRIVGIVIQMFFTLGIIILPGIAYFIPNWQGIQLAITLPSFLFLLYYWVVPESPRW 294
ICH2
ICH3
ICH4
ICH5
TM7
360 364 365
URAT1EM 283 LIIKGKPDQALQELRKVARINGHKEAKN-LTIEVLMSSVKEEVASAKEPRSVLDLFCVPGLRFRTCISTLCWFAFGFTFFGLALDLQALGSNIFLLQMFI 381
URAT1(SLC22A12) 286 LLTTGRLDWGLQELWRVAAINGKGAVQDTLTPEVLLSAMREELSMGQPPASLGTLLRMPGLRFRTCISTLCWFAFGFTFFGLALDLQALGSNIFLLQMFI 385
OAT4 (SLC22A11) 283 LIIKGKPDQALQELRKVARINGHKEAKN-LTIEVLMSSVKEEVASAKEPRSVLDLFCVPVLRWRSCAMLVVNFSLLISYYGLVFDLQSLGRDIFLLQALF 381
OAT1 (SLC22A6) 275 HSSSGRLDLTLRALQRVARINGKREEGAKLSMEVLRASLQKELTMGKGQASAMELLRCPTLRHLFLCLSMLWFATSFAYYGLVMDLQGFGVSIYLIQVIF 374
OCT1 (SLC22A1) 289 LLSQKRNTEAIKIMDHIAQKNGKLPPAD-LKMLS-----LEEDVTEKLSPSFADLFRTPRLRKRTFILMYLWFTDSVLYQGLILHMGATSGNLYLDFLYS 382
OCT3 (SLC22A3) 295 LITRKKGDKALQILRRIAKCNGKYLSSN-YSEIT-------VTDEEVSNPSFLDLVRTPQMRKCTLILMFAWFTSAVVYQGLVMRLGIIGGNLYIDFFIS 386
TM8
TM9
TM10
TM11
393
449
477
URAT1EM 382 GVVDIPAKMGALLLLSHLGRRPTLAASLLLAGLCILANTLVPHEMGALRSALAVLGLGGVGAAFTCITIYSSELFPTVLRMTAVGLGQMAARGGAILGPL 481
URAT1(SLC22A12) 386 GVVDIPAKMGALLLLSHLGRRPTLAASLLLAGLCILANTLVPHEMGALRSALAVLGLGGVGAAFTCITIYSSELFPTVLRMTAVGLGQMAARGGAILGPL 485
OAT4 (SLC22A11) 382 GAVDFLGRATTALLLSFLGRRTIQAGSQAMAGLAILANMLVPQDLQTLRVVFAVLGKGCFGISLTCLTIYKAELFPTPVRMTADGILHTVGRLGAMMGPL 481
OAT1 (SLC22A6) 375 GAVDLPAKLVGFLVINSLGRRPAQMAALLLAGICILLNGVIPQDQSIVRTSLAVLGKGCLAASFNCIFLYTGELYPTMIRQTGMGMGSTMARVGSIVSPL 474
OCT1 (SLC22A1) 383 ALVEIPGAFIALITIDRVGRIYPMAMSNLLAGAACLVMIFISPDLHWLNIIIMCVGRMGITIAIQMICLVNAELYPTFVRNLGVMVCSSLCDIGGIITPF 482
OCT3 (SLC22A3) 387 GVVELPGALLILLTIERLGRRLPFAASNIVAGVACLVTAFLPEGIAWLRTTVATLGRLGITMAFEIVYLVNSELYPTTLRNFGVSLCSGLCDFGGIIAPF 486
TM12
ICH6
URAT1EM 482 VR-LLGVHGPWLPLLVYGTVPVLSGLAAL-LLPETQSLPLPDTIQDVQNQAVKKATHGTLGNSVL-KSTQF-------------------- 549
URAT1(SLC22A12) 486 VR-LLGVHGPWLPLLVYGTVPVLSGLAAL-LLPETQSLPLPDTIQDVQNQAVKKATHGTLGNSVL-KSTQF-------------------- 553
OAT4 (SLC22A11) 482 IL-MSRQALPLLPPLLYGVISIASSLVVLFFLPETQGLPLPDTIQDLESQKSTAAQGNRQEAVTV-ESTSL-------------------- 550
OAT1 (SLC22A6) 475 VS-MTAELYPSMPLFIYGAVPVAASAVTV-LLPETLGQPLPDTVQDLESRWAPTQKEAGIYPRKGKQTRQQQEHQKYMVPLQASAQEKNGL 563
OCT1 (SLC22A1) 483 IVFRLREVWQALPLILFAVLGLLAAGVTL-LLPETKGVALPETMKDAENLGRKAKPKENTIYLKV-QTSEPSGT----------------- 554
OCT3 (SLC22A3) 487 LLFRLAAVWLELPLIIFGILASICGGLVM-LLPETKGIALPETVDDVEKLGSPHSCKCGRNKKTPVSRSHL-------------------- 556

Fig. S1 Construct characterization and sequence alignment of URAT1

a Fluorescence size-exclusion chromatography of URAT1 and OAT4. **b** Representative 2D class averages of URAT1 constructs. **c** Schematic of the $URAT1_{EM}$ construct. **d** ^{14}C-urate uptake and drug inhibition of the wild-type transporter ($URAT1_{WT}$) and the cryo-EM construct ($URAT1_{EM}$). Uptake buffer: 20 mM HEPES pH 7.4, 125 mM sodium gluconate, 4.8 mM potassium gluconate, 1.2 mM monobasic potassium phosphate, 1.2 mM magnesium sulfate, 1.3 mM calcium gluconate, and 5.6 mM glucose. Graph shows individual data points, mean ± s.d.; n = 5 biological replicates. **e** Sequence alignment of the selected members from the SLC22 family. Blue shade, residues that form the phenylalanine cage; yellow shade, key gating residues that interacts with urate and anti-gout drugs. Blue box highlights the region of construct modification.
