# Supplementary material for: Transport mechanism and structural pharmacology of human urate transporter URAT1
Source: Cell Res. 2024 Sep 9;34(11):776–87. doi: 10.1038/s41422-024-01023-1 (PMC11528023; doi:10.1038/s41422-024-01023-1)
Supplement: Supplementary file 3 — Supplementary information Fig S3 [file 41422_2024_1023_MOESM3_ESM.pdf]

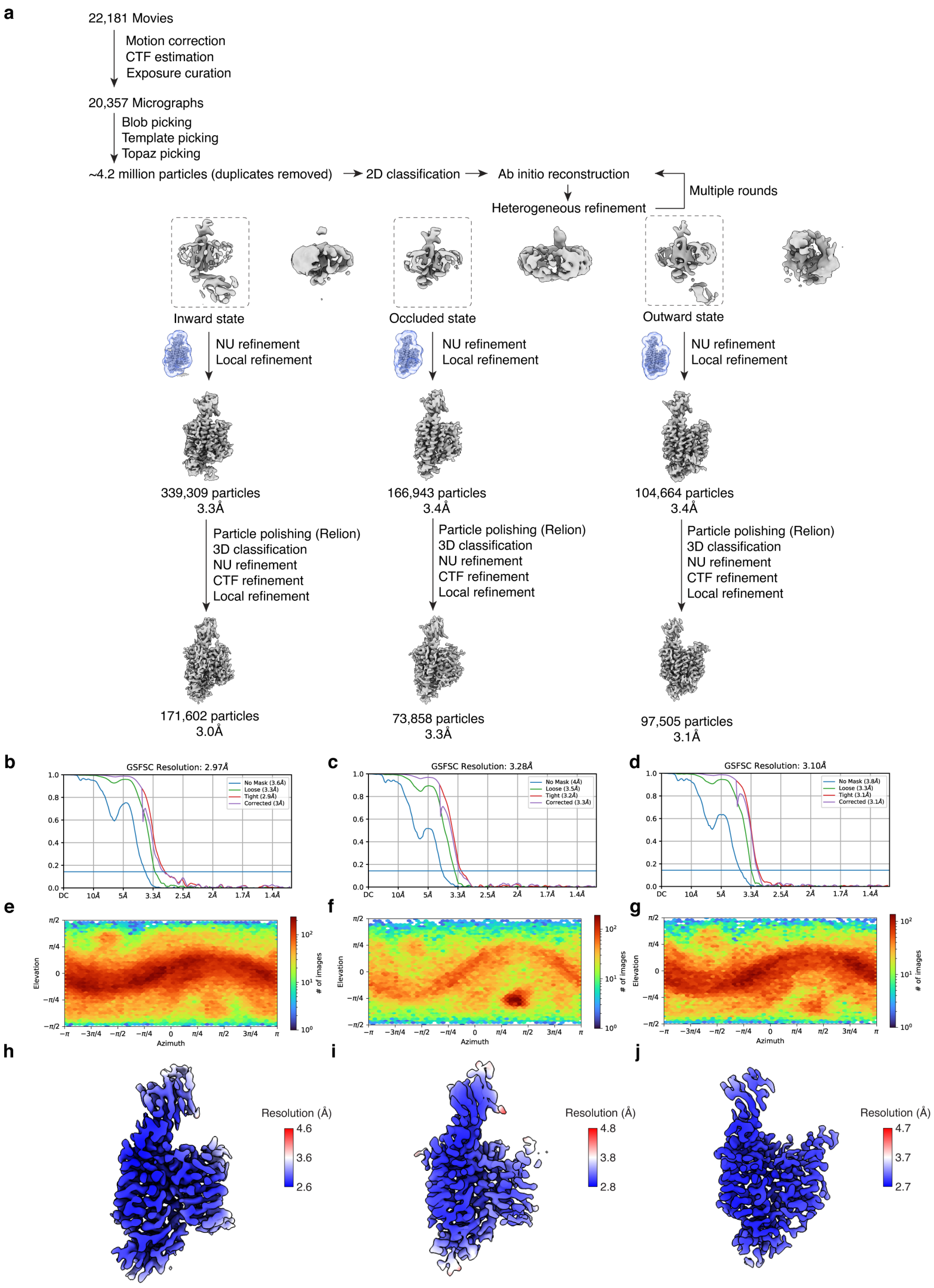
a
22,181 Movies
Motion correction
CTF estimation
Exposure curation
20,357 Micrographs
Blob picking
Template picking
Topaz picking
~4.2 million particles (duplicates removed)
2D classification
Ab initio reconstruction
Heterogeneous refinement
Multiple rounds
Inward state
Occluded state
Outward state
NU refinement
Local refinement
339,309 particles
3.3Å
166,943 particles
3.4Å
104,664 particles
3.4Å
Particle polishing (Relion)
3D classification
NU refinement
CTF refinement
Local refinement
171,602 particles
3.0Å
73,858 particles
3.3Å
97,505 particles
3.1Å
b
GSFSC Resolution: 2.97Å
No Mask (3.6Å)
Loose (3.3Å)
Tight (2.9Å)
Corrected (3Å)
c
GSFSC Resolution: 3.28Å
No Mask (4Å)
Loose (3.5Å)
Tight (3.2Å)
Corrected (3.3Å)
d
GSFSC Resolution: 3.10Å
No Mask (3.8Å)
Loose (3.3Å)
Tight (3.1Å)
Corrected (3.1Å)
DC
10Å
5Å
3.3Å
2.5Å
2Å
1.7Å
1.4Å
e
f
g
Elevation
Azimuth
# of images
h
i
j
Resolution (Å)
4.6
3.6
2.6
4.8
3.8
2.8
4.7
3.7
2.7

Fig. S3 Cryo-EM analyses of URAT1 in urate-bound states

a Summary of image processing procedures of $URAT1_{EM}$ in the presence of urate. All procedures were done with cryoSPARC, except for particle polishing which was done with RELION. **b–d** Fourier shell correlation (FSC) curves between two half maps of the inward, occluded, and outward conformations, respectively. **e–g** Angular distributions of particles for the final 3D reconstructions of the inward, occluded, and outward conformations, respectively. **h–j** Local resolution of the cryo-EM maps of the inward, occluded, and outward conformations, respectively.
