# Supplementary material for: Transport mechanism and structural pharmacology of human urate transporter URAT1
Source: Cell Res. 2024 Sep 9;34(11):776–87. doi: 10.1038/s41422-024-01023-1 (PMC11528023; doi:10.1038/s41422-024-01023-1)
Supplement: Supplementary file 4 — Supplementary information Fig S4 [file 41422_2024_1023_MOESM4_ESM.pdf]

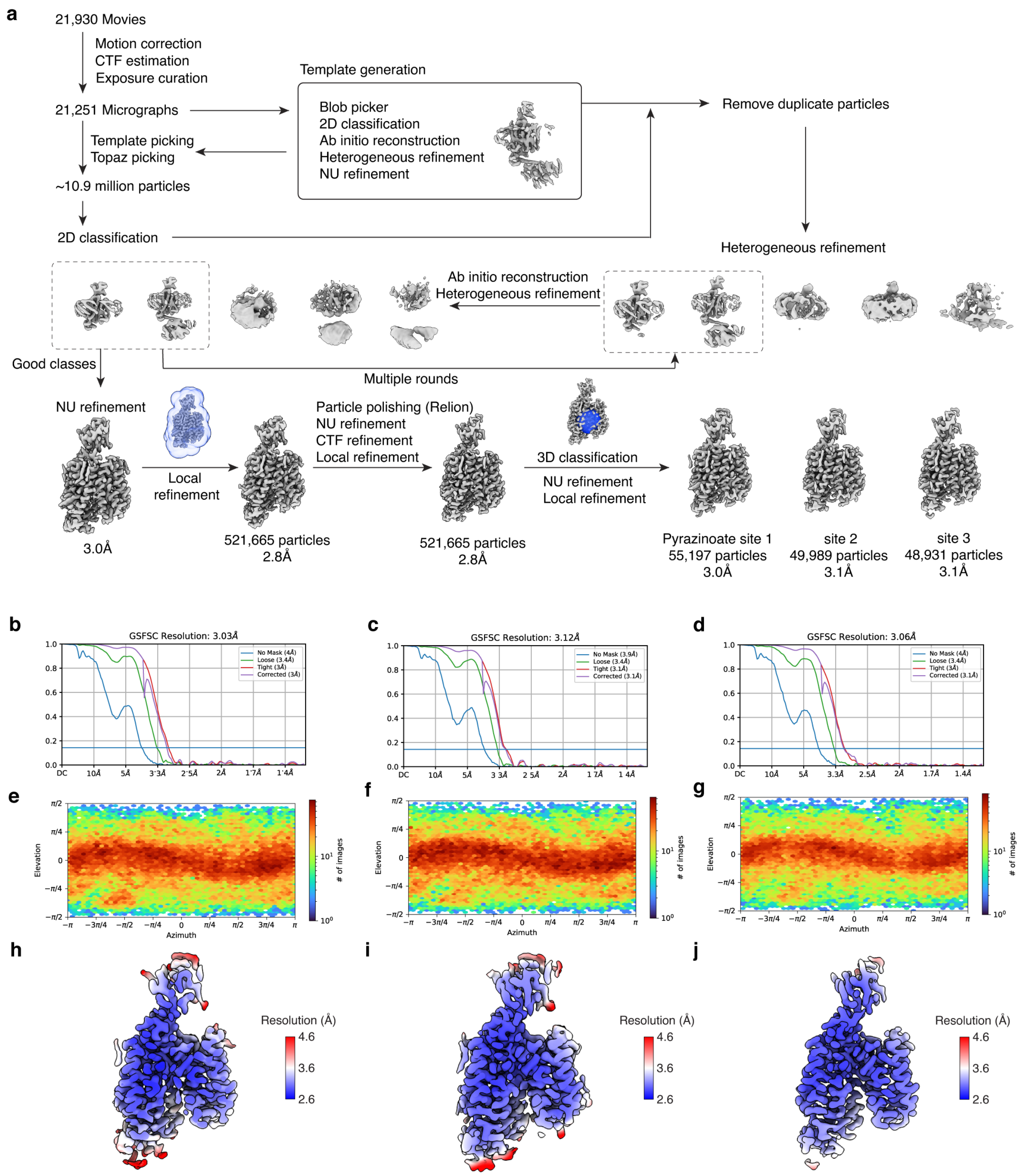

Fig. S4 Cryo-EM analyses of URAT1 in pyrazinoate-bound states

a Summary of image processing procedures of URAT1$_{EM}$ in the presence of pyrazinoate. All procedures were done with cryoSPARC, except for particle polishing which was done with RELION. **b–d** Fourier shell correlation (FSC) curves between two half maps of the

pyrazinoate Site 1-, Site 2-, and Site 3-bound conformations, respectively. **e–g** Angular distributions of particles for the final 3D reconstructions of the pyrazinoate Site 1-, Site 2-, and Site 3-bound conformations, respectively. **h–j** Local resolution of the cryo-EM maps of the pyrazinoate Site 1-, Site 2-, and Site 3-bound conformations, respectively.
