# Supplementary material for: Transport mechanism and structural pharmacology of human urate transporter URAT1
Source: Cell Res. 2024 Sep 9;34(11):776–87. doi: 10.1038/s41422-024-01023-1 (PMC11528023; doi:10.1038/s41422-024-01023-1)
Supplement: Supplementary file 5 — Supplementary information Fig S5 [file 41422_2024_1023_MOESM5_ESM.pdf]

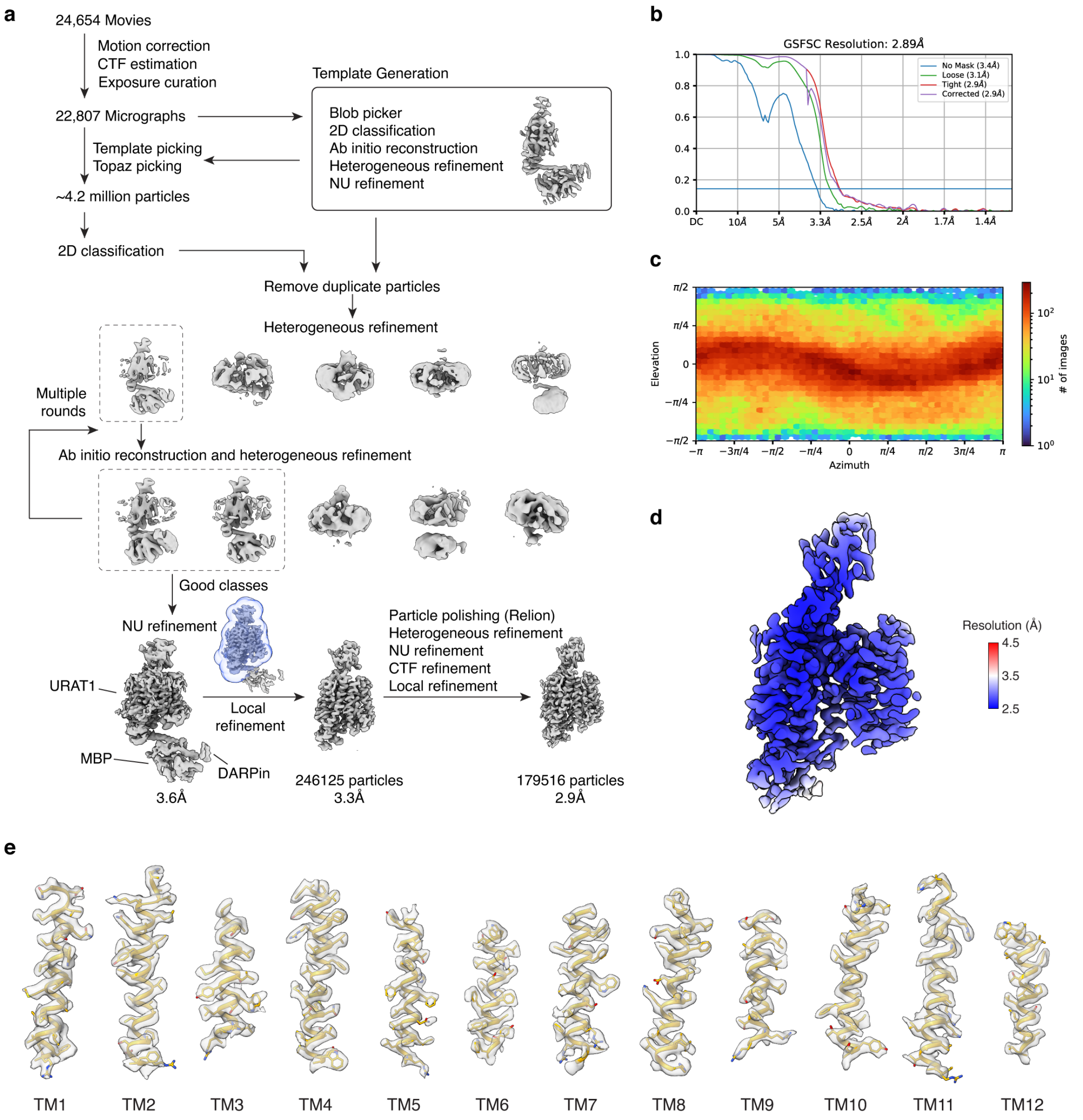

Fig. S5 Cryo-EM analyses of URAT1 in the lesinurad-bound state

a Summary of image processing procedures of URAT1$_{EM}$ in the presence of lesinurad. All procedures were done with cryoSPARC, except for particle polishing which was done with RELION. **b** Fourier shell correlation (FSC) curves between two half maps. **c** Angular distribution of particles for the final 3D reconstructions. **d** Local resolution of the cryo-EM map. **e** Cryo-EM densities of the transmembrane helices.
