# Supplementary material for: Transport mechanism and structural pharmacology of human urate transporter URAT1
Source: Cell Res. 2024 Sep 9;34(11):776–87. doi: 10.1038/s41422-024-01023-1 (PMC11528023; doi:10.1038/s41422-024-01023-1)
Supplement: Supplementary file 8 — Supplementary information Fig S8 [file 41422_2024_1023_MOESM8_ESM.pdf]

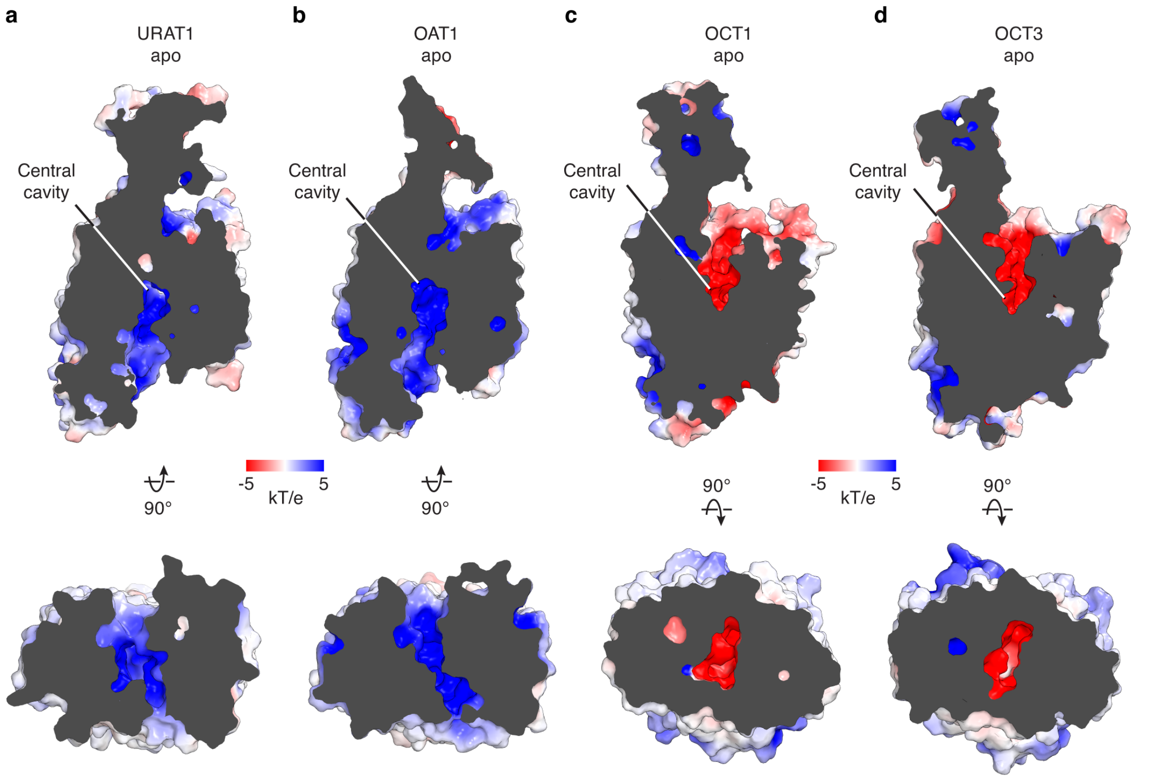

Fig. S8 Electrostatic properties of SLC22 transporters in the apo state

a Human URAT1. **b** Rat OAT1 (PDB: 8BVR). **c** Human OCT1 (PDB: 8ET6). **d** Human OCT3 (PDB: 7ZH0). The electrostatic surface potential is calculated with the program APBS (http://www.poissonboltzmann.org).
