# Supplementary material for: Transport mechanism and structural pharmacology of human urate transporter URAT1
Source: Cell Res. 2024 Sep 9;34(11):776–87. doi: 10.1038/s41422-024-01023-1 (PMC11528023; doi:10.1038/s41422-024-01023-1)
Supplement: Supplementary file 9 — Supplementary information Fig S9 [file 41422_2024_1023_MOESM9_ESM.pdf]

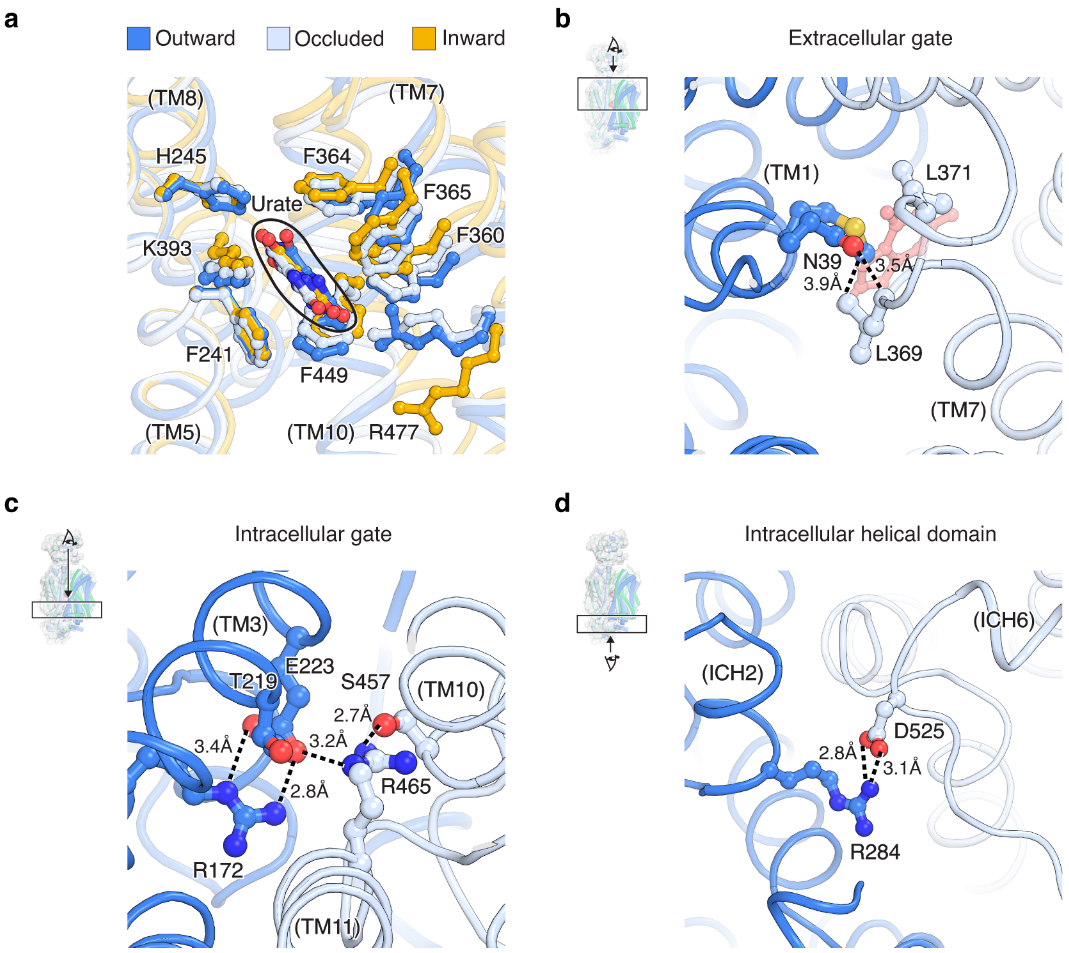

Fig. S9 Structural analyses of the urate pocket and transporter gates

a Superimposition of urate-bound URAT1 in the outward-facing, occluded, and inward-facing conformations, showing the local rearrangements of the urate pocket. **b** Detailed interactions in the extracellular gate. The occluded URAT1 structure is shown. **c** Detailed interactions in the intracellular gate. **d** Detailed interactions in the intracellular helical domain. In **c** and **d** the outward-facing structure is shown.
