# Supplementary material for: Transport mechanism and structural pharmacology of human urate transporter URAT1
Source: Cell Res. 2024 Sep 9;34(11):776–87. doi: 10.1038/s41422-024-01023-1 (PMC11528023; doi:10.1038/s41422-024-01023-1)
Supplement: Supplementary file 10 — Supplementary information Fig S10 [file 41422_2024_1023_MOESM10_ESM.pdf]

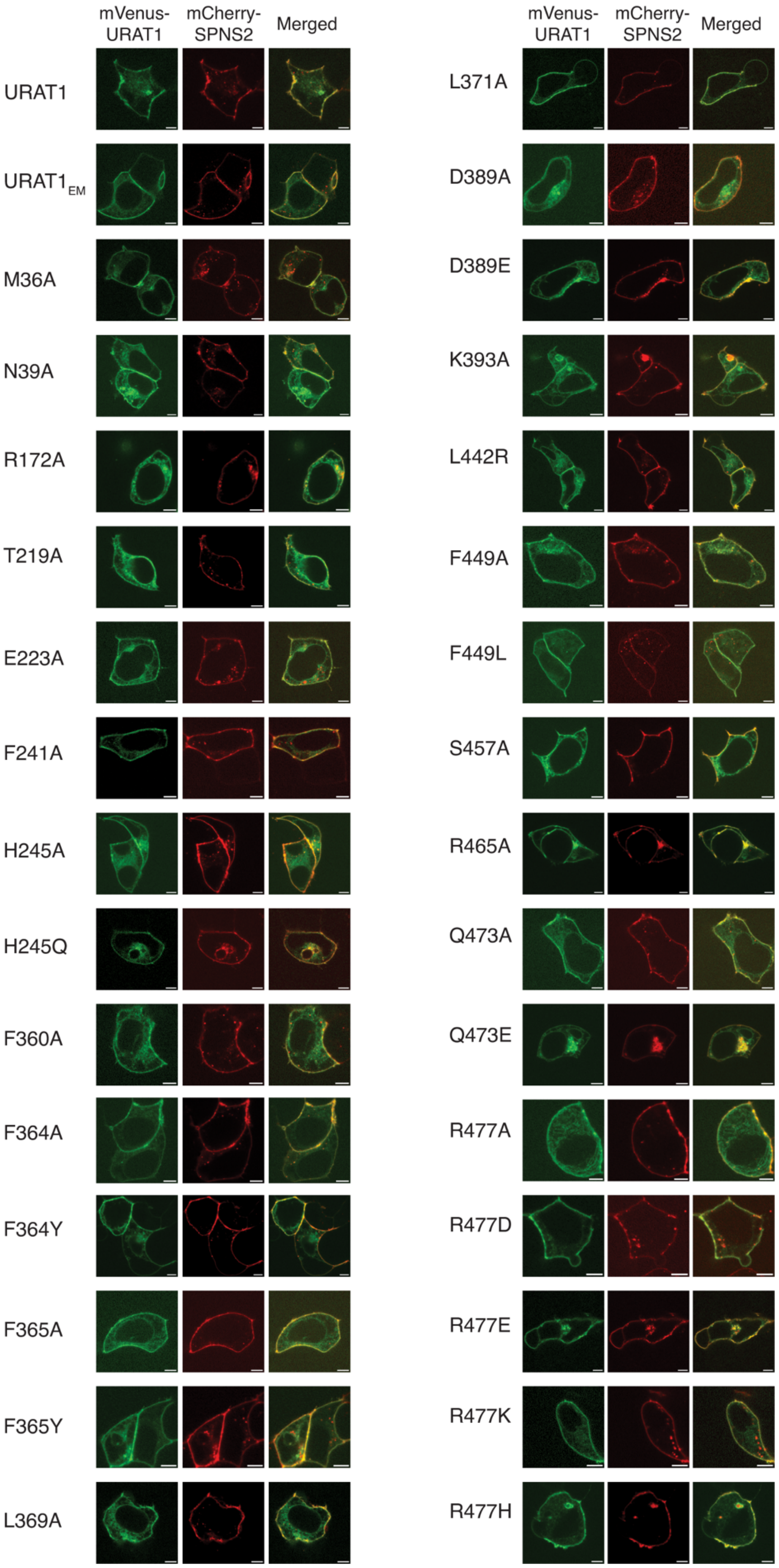
mVenus-URAT1
mCherry-SPNS2
Merged
URAT1
URAT1$_{EM}$
M36A
N39A
R172A
T219A
E223A
F241A
H245A
H245Q
F360A
F364A
F364Y
F365A
F365Y
L369A
L371A
D389A
D389E
K393A
L442R
F449A
F449L
S457A
R465A
Q473A
Q473E
R477A
R477D
R477E
R477K
R477H

Fig. S10 Trafficking of URAT1 variants

Representative images of the cellular localization of URAT1 in HEK293 cells. Plasma membrane marker, Spinster homolog 2 (Spns2).
