# Supplementary material for: Transport mechanism and structural pharmacology of human urate transporter URAT1
Source: Cell Res. 2024 Sep 9;34(11):776–87. doi: 10.1038/s41422-024-01023-1 (PMC11528023; doi:10.1038/s41422-024-01023-1)
Supplement: Supplementary file 11 — Supplementary information Fig S11 [file 41422_2024_1023_MOESM11_ESM.pdf]

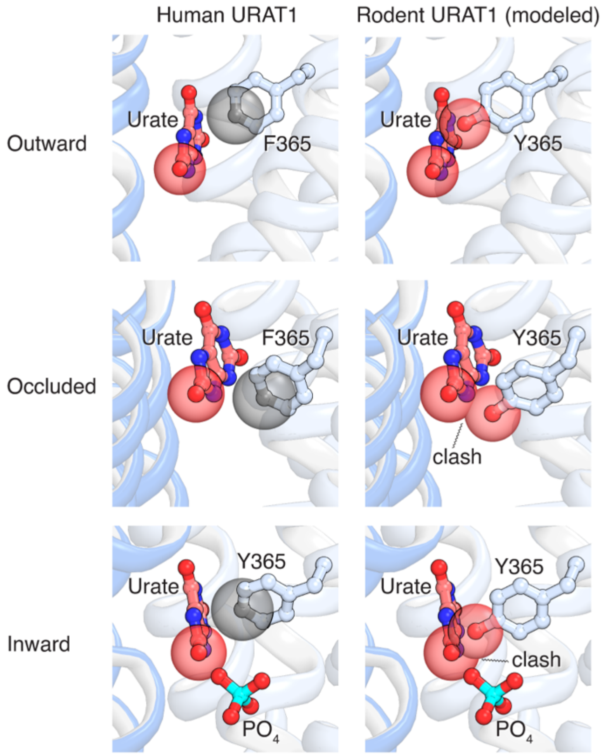

Fig. S11 Potential steric clashes between URAT1 F365Y and urate

Tyrosine at residue 365 in rats or mice may cause steric hindrance against urate.
