# Supplementary material for: Transport mechanism and structural pharmacology of human urate transporter URAT1
Source: Cell Res. 2024 Sep 9;34(11):776–87. doi: 10.1038/s41422-024-01023-1 (PMC11528023; doi:10.1038/s41422-024-01023-1)
Supplement: Supplementary file 12 — Supplementary information Fig S12 [file 41422_2024_1023_MOESM12_ESM.pdf]

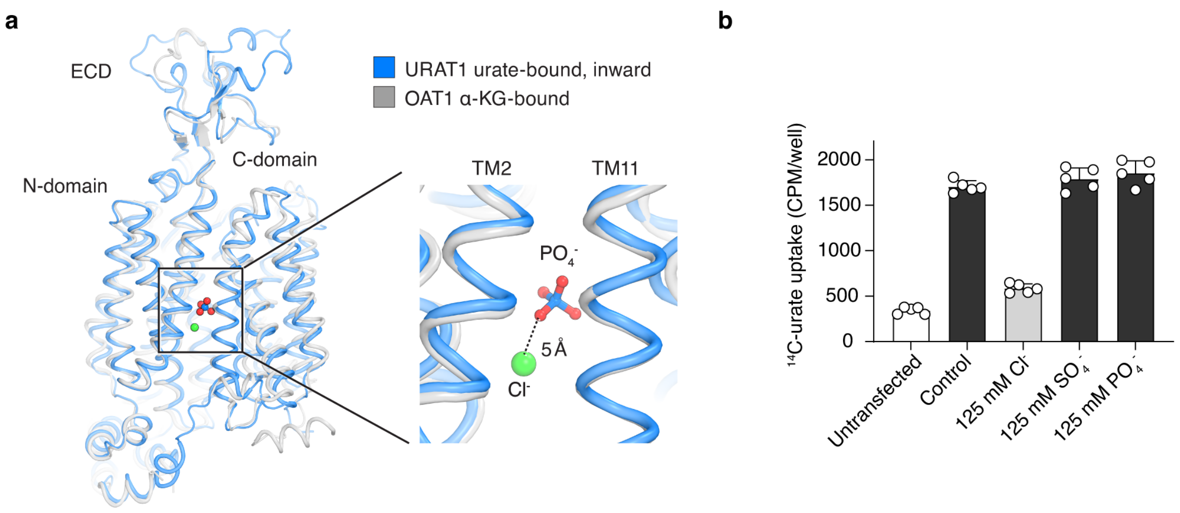

Fig. S12 Putative phosphate binding site in URAT1

a Comparison of the urate-bound, inward-facing conformation of URAT1 with the α-ketoglutarate-bound conformation of OAT1 (PDB: 8BW7). **b** Effect of reducing anion gradients (intracellular to extracellular) on ^{14}C-urate uptake. Uptake buffer: 20 mM HEPES pH 7.4, 125 mM sodium gluconate, 5.6 mM glucose (control); 20 mM HEPES pH 7.4, 125 mM sodium chloride, 5.6 mM glucose; 20 mM HEPES pH 7.4, 125 mM sodium sulfate, 5.6 mM glucose; 20 mM HEPES pH 7.4, 125 mM sodium phosphate, 5.6 mM glucose. Graph shows individual data points, mean ± s.d.; n = 5 biological replicates.
