# Supplementary material for: Transport mechanism and structural pharmacology of human urate transporter URAT1
Source: Cell Res. 2024 Sep 9;34(11):776–87. doi: 10.1038/s41422-024-01023-1 (PMC11528023; doi:10.1038/s41422-024-01023-1)
Supplement: Supplementary file 13 — Supplementary information Fig S13 [file 41422_2024_1023_MOESM13_ESM.pdf]

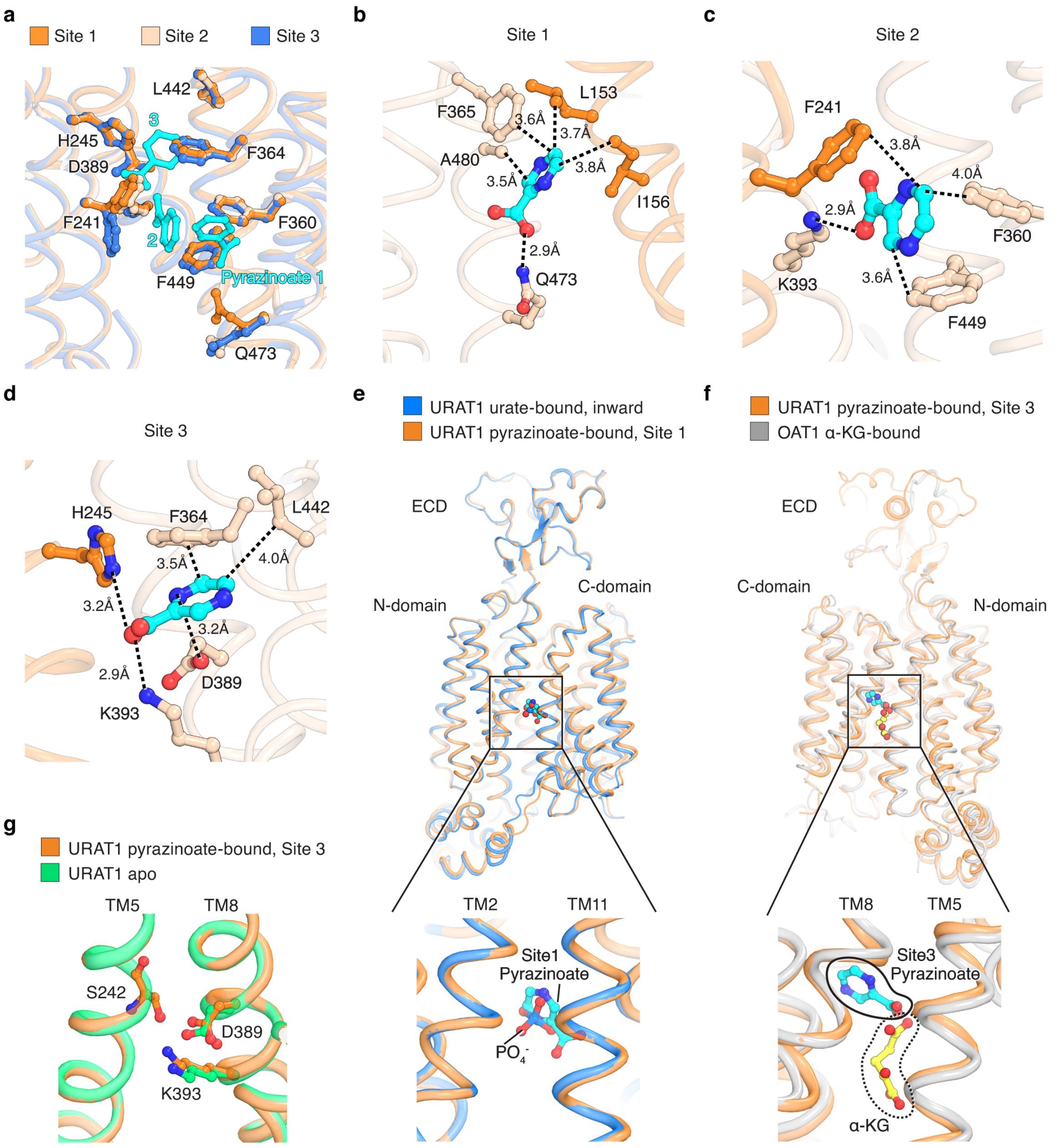

Fig. S13 Structural analyses of the pyrazinoate sites

a Superimposition of the pyrazinoate-bound URAT1 structures, showing local rearrangements of the residues lining the pyrazinoate-binding sites. **b–d** Detailed interactions of pyrazinoate in Site 1, 2, and 3, respectively. **e** Comparison between the urate-bound, inward-facing conformation with the Site 1 pyrazinoate-bound conformation of URAT1. **f** Comparison of the Site 3 pyrazinoate-bound conformation of URAT1 with the α-ketoglutarate-bound conformation of OAT1 (PDB: 8BW7). **g** Comparison between the Site 3 pyrazinoate-bound conformation with the apo conformation of URAT1.
