# Supplementary material for: Transport mechanism and structural pharmacology of human urate transporter URAT1
Source: Cell Res. 2024 Sep 9;34(11):776–87. doi: 10.1038/s41422-024-01023-1 (PMC11528023; doi:10.1038/s41422-024-01023-1)
Supplement: Supplementary file 14 — Supplementary information Fig S14 [file 41422_2024_1023_MOESM14_ESM.pdf]

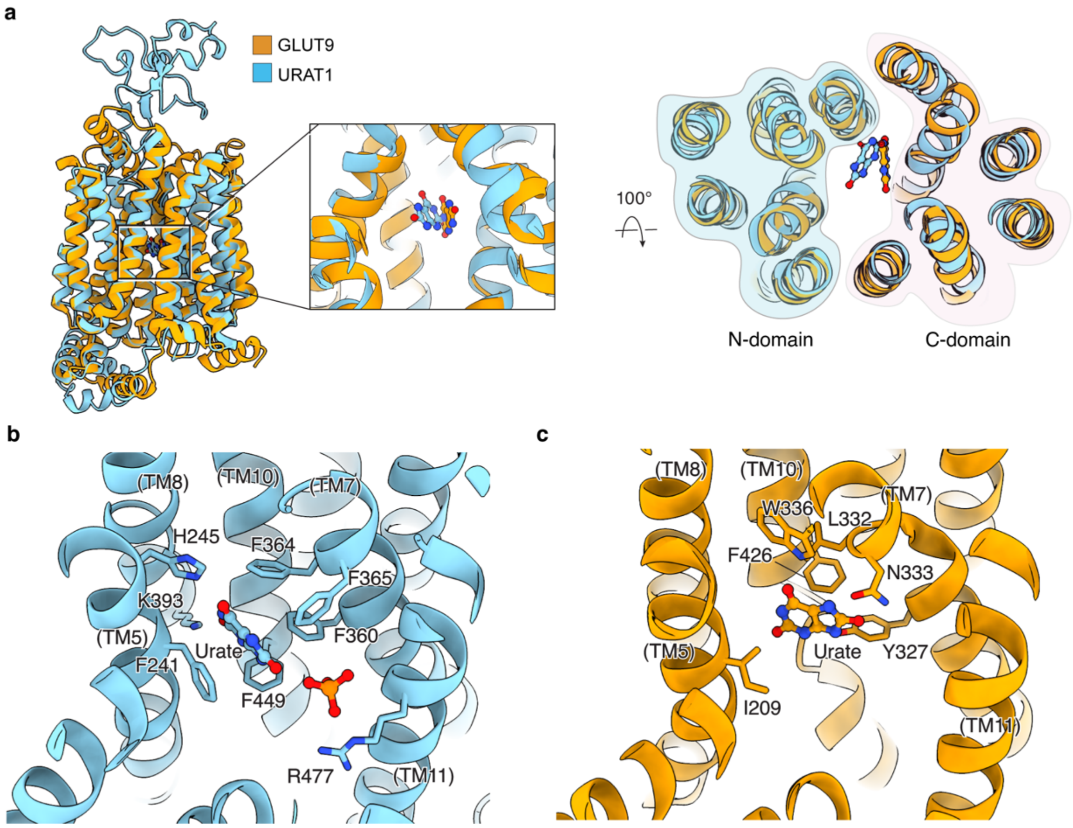

Fig. S14 Urate recognition in URAT1 and GLUT9

a Superimposition of URAT1 and GLUT9 in their respective urate-bound, inward-facing conformations. **b** and **c** Comparison of the detailed interactions within the urate pockets of URAT1 and GLUT9 (PDB: 8Y65).
