# Supplementary material for: Transport mechanism and structural pharmacology of human urate transporter URAT1
Source: Cell Res. 2024 Sep 9;34(11):776–87. doi: 10.1038/s41422-024-01023-1 (PMC11528023; doi:10.1038/s41422-024-01023-1)
Supplement: Supplementary file 15 — Supplementary information Fig S15 [file 41422_2024_1023_MOESM15_ESM.pdf]

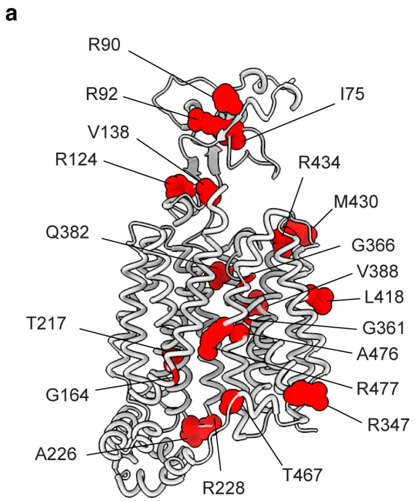

b

Variants	Functional validation
I75T	Reduced urate transport, impaired membrane trafficking
R90H	Reduced urate transport
R92C	Not tested
R124L	Reduced urate transport
V138M	Reduced urate transport
G164S	Reduced urate transport
T217M	Reduced urate transport
A226V	Not tested
R228E	No effect on urate transport
R284G	Not tested
G290C	Not tested
Q297E	Not tested
E298D	Reduced urate transport
I305S	Not tested
Q312L	Not tested
R347S	Reduced urate transport
G361V	Reduced urate transport
G366R	Reduced urate transport, impaired membrane trafficking
Q382L	Reduced urate transport
V388M	No effect on urate transport, impaired membrane trafficking
L418R	Reduced urate transport
M430T	Reduced urate transport
R434C	Reduced urate transport, impaired membrane trafficking
R434H	Reduced urate transport, impaired membrane trafficking
T467M	Reduced urate transport, impaired membrane trafficking
A476D	Reduced urate transport
R477H	Reduced urate transport
R477S	Reduced urate transport

Fig. S15 Locations of disease-related URAT1 mutations

a Human disease mutations mapped on the apo structure of URAT1. Note that some mutations cannot be mapped because they are located in the intracellular helical domain, which is modified in the $URAT1_{EM}$ construct. **b** Functional consequences of disease mutations. The phenotypes summarized here are from previous literature [6,9–12,43–48] and the current study.
