# Supplementary material for: Transport mechanism and structural pharmacology of human urate transporter URAT1
Source: Cell Res. 2024 Sep 9;34(11):776–87. doi: 10.1038/s41422-024-01023-1 (PMC11528023; doi:10.1038/s41422-024-01023-1)
Supplement: Supplementary file 16 — Supplementary information Table S1 [file 41422_2024_1023_MOESM16_ESM.pdf]

Structure	Apo	Urate-bound outward-facing	Urate-bound occluded	Urate-bound inward-facing	Pyrazinoate-bound Site1
PDB	9B1F	9B1L	9B1K	9B1J	9B1M
EMDB	44077	44083	44082	44081	44084
**Data collection/ processing**					
Magnification	130,000x	130,000x	130,000x	130,000x	130,000x
Voltage (kV)	300	300	300	300	300
Pixel size (Å)	0.649	0.649	0.649	0.649	0.649
Defocus range (μm)	0.6-1.6	1.1-2.1	1.1-2.1	1.1-2.1	1.1-2.1
Electron exposure (e^-/Å^2)	70.6	72.8	72.8	72.8	68.8
Symmetry imposed	C1	C1	C1	C1	C1
Initial particles (No.)	~4.5 millions	~4.2 millions	~4.2 millions	~4.2 millions	~10.9 millions
Final particles (No.)	328,579	97,505	73,858	171,602	55,197
Map resolution (Å)	2.90	3.10	3.28	2.97	3.03
FSC threshold	0.143	0.143	0.143	0.143	0.143
**Refinement**					
Model composition					
Non-hydrogen atoms	3937	4041	3911	3954	3962
Protein residues	515	529	512	515	515
Ligand	NAG:1	URATE:1	URATE:1	URATE:1<br>NAG:1<br>PO_4:1	NAG:1<br>POA:1
B-factors (Å^2)					
Protein	52.33	84.54	78.43	77.44	115.12
Ligand	105.71	78.65	73.47	101.95	151.51
R.m.s. deviations					
Bond lengths (Å)	0.004	0.004	0.004	0.004	0.004
Bond angles (°)	0.608	0.659	0.594	0.705	0.763
Validation					
MolProbity score	0.87	0.67	0.85	0.8	0.71
Clashscore	1.38	0.49	1.26	1.00	0.62
Rotamers outliers (%)	0.00	0.00	0.00	0.00	0.00
Ramachandran plot (%)					
Favored	98.83	98.67	98.24	98.83	98.83
Allowed	1.17	1.33	1.76	1.17	1.17
Outliers	0.00	0.00	0.00	0.00	0.00

Structure	Pyrazinoate-bound Site2	Pyrazinoate-bound Site3	Lesinurad-bound	Verinurad-bound	Dotinurad-bound
PDB	9B1N	9B1O	9B1H	9B1I	9B1G
EMDB	44085	44086	44079	44080	44078
**Data collection/ processing**					
Magnification	130,000x	130,000x	130,000x	130,000x	130,000x
Voltage (kV)	300	300	300	300	300
Pixel size (Å)	0.649	0.649	0.649	0.649	0.649
Defocus range (μm)	1.1-2.1	1.1-2.1	0.6-1.6	0.6-1.6	0.6-1.6
Electron exposure (e^-/Å^2)	68.8	68.8	71.9	77.6	68.1
Symmetry imposed	C1	C1	C1	C1	C1
Initial particles (No.)	~10.9 millions	~10.9 millions	~4.2 millions	~5.8 millions	~4.5 millions
Final particles (No.)	49,989	48931	179,516	220,155	199,921
Map resolution (Å)	3.12	3.06	2.89	3.73	2.70
FSC threshold	0.143	0.143	0.143	0.143	0.143
**Refinement**					
Model composition					
Non-hydrogen atoms	3955	3946	3961	3962	3959
Protein residues	515	515	515	515	515
Ligand	NAG:1	NAG:1	LES:1	VER:1	DOT:1
	POA:1	POA:1	NAG:1	NAG:1	NAG:1
B-factors (Å^2)					
Protein	72.97	90.72	85.64	54.78	58.56
Ligand	106.29	130.00	110.07	59.14	76.16
R.m.s. deviations					
Bond lengths (Å)	0.005	0.004	0.004	0.003	0.003
Bond angles (°)	0.786	0.761	0.655	0.617	0.589
Validation					
MolProbity score	0.74	0.71	0.85	0.74	0.85
Clashscore	0.75	0.63	1.25	0.75	1.25
Rotamers outliers (%)	0.00	0.00	0.00	0.00	0.00
Ramachandran plot (%)					
Favored	98.44	98.44	99.22	98.44	99.22
Allowed	1.56	1.56	0.78	1.56	0.78
Outliers	0.00	0.00	0.00	0.00	0.00

Table S1 Cryo-EM data collection, processing, and refinement statistics
